# A mixed methods approach identifying facilitators and barriers to guide adaptations to InterCARE strategies: an integrated HIV and hypertension care model in Botswana

**DOI:** 10.1186/s43058-024-00603-x

**Published:** 2024-06-20

**Authors:** Pooja Gala, Ponego Ponatshego, Laura M. Bogart, Nabila Youssouf, Mareko Ramotsababa, Amelia E. Van Pelt, Thato Moshomo, Evelyn Dintwa, Khumo Seipone, Maliha Ilias, Veronica Tonwe, Tendani Gaolathe, Lisa R. Hirschhorn, Mosepele Mosepele

**Affiliations:** 1 Department of Medicine, NYU Langone Grossman School of Medicine, New York, NY USA; 2 Department of Internal Medicine, Faculty of Medicine, University of Botswana, Gaborone, Botswana; 3 Botswana Harvard AIDS Institute Partnership, Gaborone, Botswana; 4 RAND Corporation, Santa Monica, CA USA; 5 Department of Clinical Research, Faculty of Infectious and Tropical Diseases, London School of Hygiene and Tropical Medicine, London, UK; 6 Government of Botswana, Ministry of Health and Wellness, Gaborone, Botswana; 7 Department of Medical Social Sciences, Northwestern University, Chicago, IL USA; 8 Center for Translation Research and Implementation Science, Department of Health and Human Services, National Heart, Lung and Blood Institute, National Institutes of Health, Bethesda, MD USA

**Keywords:** HIV, Hypertension, Low- and middle- income countries, Integrated care, Botswana, Implementation science

## Abstract

**Background:**

Botswana serves as a model of success for HIV with 95% of people living with HIV (PLWH) virally suppressed. Yet, only 19% of PLWH and hypertension have controlled blood pressure. To address this gap, InterCARE, a care model that integrates HIV and hypertension care through a) provider training; b) adapted electronic health record; and c) treatment partners (peer support), was designed. This study presents results from our baseline assessment of the determinants and factors used to guide adaptations to InterCARE implementation strategies prior to a hybrid type 2 effectiveness-implementation study.

**Methods:**

This study employed a convergent mixed methods design across two clinics (one rural, one urban) to collect quantitative and qualitative data through facility assessments, 100 stakeholder surveys (20 each PLWH and hypertension, existing HIV treatment partners, clinical healthcare providers (HCPs), and 40 community leaders) and ten stakeholder key informative interviews (KIIs). Data were analyzed using descriptive statistics and deductive qualitative analysis organized by the Consolidated Framework for Implementation Research (CFIR) and compared to identify areas of convergence and divergence.

**Results:**

Although 90.3% of 290 PLWH and hypertension at the clinics were taking antihypertensive medications, 52.8% had uncontrolled blood pressure. Results from facility assessments, surveys, and KIIs identified key determinants in the CFIR innovation and inner setting domains. Most stakeholders (> 85%) agreed that InterCARE was adaptable, compatible and would be successful at improving blood pressure control in PLWH and hypertension. HCPs agreed that there were insufficient resources (40%), consistent with facility assessments and KIIs which identified limited staffing, inconsistent electricity, and a lack of supplies as key barriers. Adaptations to InterCARE included a task-sharing strategy and expanded treatment partner training and support.

**Conclusions:**

Integrating hypertension services into HIV clinics was perceived as more advantageous for PLWH than the current model of hypertension care delivered outside of HIV clinics. Identified barriers were used to adapt InterCARE implementation strategies for more effective intervention delivery.

**Trial registration:**

ClinicalTrials.gov, ClinicalTrials.gov Identifier: NCT05414526. Registered 18 May 2022 – Retrospectively registered.

**Supplementary Information:**

The online version contains supplementary material available at 10.1186/s43058-024-00603-x.

Contributions to the literature

- Using existing HIV infrastructure through the integration of services is an intervention strategy that is gaining traction worldwide to address co-morbid hypertension and non-communicable diseases.
- The Consolidated Framework for Implementation Research (CFIR) can be utilized to identify the determinants of implementation and guide adaptations of implementation strategies for integrated care.
- We found that available resources and levels of staffing were significant barriers to intervention implementation, addressed by stakeholder engagement and a nurse task shifting strategy.
- When developing a model of integrated care, including the perspectives of multiple key stakeholder groups is crucial to identifying determinants of implementation and adapting integration strategies.

## Background

The global availability of effective treatment for HIV has transformed HIV into a chronic condition. In sub-Saharan Africa, home to a third of global HIV infections, effective healthcare delivery models have resulted in multiple countries, including Botswana [1, 2], approaching or achieving key global targets for the HIV care cascade: 95% aware of their diagnosis, 95% on antiretroviral therapy, and 95% with viral suppression [1, 3]. In Botswana, where in 2017 an estimated 20.3% of the adult population had HIV [4], factors contributing to achieving these targets include having specialized HIV clinics [5], trained healthcare providers (HCPs) in HIV care, task shifting (e.g., licensing antiretroviral therapy nurse prescribers) [6, 7], using electronic health records [8], making HIV medications free and accessible, and using treatment partners (i.e., participant-chosen peers who counsel and support people living with HIV (PLWH) in attending clinic appointments and taking their medications) [9, 10].

As the survival of PLWH has improved, PLWH are developing other chronic conditions, such as cardiovascular disease (CVD). PLWH are twice as likely to develop CVD than people without HIV infection [11, 12]. Hypertension is a leading risk factor for CVD in the general population and among PLWH globally [4, 13–16]. A recent hypertension study nested within the Botswana HIV Combination Prevention Project found that nearly one-third of PLWH had hypertension [4]. Amongst these individuals, only 46.0% were aware of their hypertension diagnosis, and 42% of those aware were on hypertension treatment; 44% of those on treatment had controlled blood pressure, resulting in only 19% of all people living with HIV and with hypertension (PLWH and hypertension) in the study having attained blood pressure control [4].

Recently, the country’s focus has expanded from HIV alone to co-morbid disease management (e.g., hypertension) amongst PLWH. Building on the successes of HIV care, one evidence-based intervention to improving hypertension management is the integration of hypertension prevention and treatment into existing longitudinal models of HIV care [17–19]. To address the gap in hypertension care for PLWH in Botswana, a bundle of strategies to achieve hypertension and HIV integration was designed: Integrating hypertension and Cardiovascular Care into Existing HIV Services Package in Botswana (InterCARE). This bundle involves hypertension care integrated into the HIV clinic supported by three evidence-based strategies: a) adapted Electronic Health Record to capture hypertension risk and treatment, b) provider training on hypertension diagnosis and management, and c) treatment partners to support adherence to hypertension care and treatment. While there is evidence of the effectiveness of these implementation strategies in HIV clinics in increasing uptake of antiretroviral therapy [6, 7], there is less evidence on the acceptability of, appropriateness of, and factors affecting strategies for integrated hypertension and HIV care in Botswana. This study aimed to identify gaps in current care, summarize the factors affecting the implementation of InterCARE, and highlight the adaptations made to InterCARE implementation strategies prior to the pilot study. The results provided valuable insight into the determinants of implementation that informed tailoring of the InterCARE intervention to the local context prior to a two-stage type 2 hybrid effectiveness-implementation trial.

## Methods

### Overview of study design and methods

The InterCARE intervention trial is a two-stage type 2 hybrid effectiveness-implementation cluster randomized control trial of a multi-component, multi-level implementation intervention aimed at reducing the risk of CVD among adults with a dual diagnosis of HIV and hypertension followed in HIV clinics in Botswana. The first stage is a pilot study assessing the feasibility of implementing InterCARE. Prior to the pilot study, formative work was conducted to measure gaps in care, facility readiness, and factors affecting the implementation of InterCARE. These results informed adaptations to the InterCARE implementation strategies prior to the pilot study. We present a mixed methods convergent analysis of this formative work and describe the resulting adaptations.

### Study setting

The HIV/Infectious Diseases Care Clinic (IDCC) model of care in Botswana, established in 2001 in response to the HIV pandemic, is a HIV clinic for PLWH that provides services limited to HIV management including antiretroviral therapy initiation, follow-up, and antiretroviral therapy failure management [5]. PLWH and hypertension separately attend the general clinic for the management of their hypertension and the remainder of their other healthcare conditions. Two public HIV clinics were chosen for pre-implementation data collection and the pilot study including a small clinic (S clinic) in the south of Botswana (*n =* 500 patients, rural, staffed primarily by nurses) and a large community clinic (L clinic) in the northeast of Botswana (*n* ≥ 3,000 patients, urban, staffed by nurses, doctors and a family nurse practitioner (FNP)). The clinics were chosen to optimize variability in the level of care and size across clinics in the primary health HIV care model in Botswana.

### Study population

#### Eligibility and recruitment

##### Baseline population

PLWH and hypertension (prior diagnosis or newly diagnosed hypertension defined as blood pressure ≥ 140/90 at baseline study visit) aged 20–75 receiving HIV care at one of the two study sites were consecutively enrolled between October 2021 to November 2021 until a sample size of 290 individuals was achieved (*n =* 50 in S clinic and *n =* 240 in L clinic).

##### Pre-implementation stakeholders

Individuals were eligible to complete pre-implementation surveys based on the following criteria for each of the four groups: 1) all HCPs at both clinics, 2) PLWH and hypertension aged 20–75 receiving HIV care at one of the two study sites, 3) HIV treatment partners (e.g., participant-chosen peers who counsel and support PLWH in attending clinic appointments and taking their medications) aged 20–75 who were actively supporting PLWH at one of the two study clinics, and 4) community members (e.g., local leaders, local council members and leaders in the commercial sector) over the age of 20 who attended one of the two study. PLWH and hypertension (*n =* 20, 50% female), HIV treatment partners (*n =* 20, 95% female) and HCPs (*n =* 20, 55% female) were consecutively recruited in person at each clinic site for the pre-implementation surveys and community members (*n =* 40, 57.5% female) were purposively selected based on role in the community until the targeted number was reached (Table 1, Supplemental Materials).

For key informant interviews (KIIs), from the stakeholders surveyed, a diverse sample of stakeholders with a wide range of perspectives were purposively selected based on their clinic location, and availability to be interviewed (*n =* 2 HCPs, *n =* 3 community members, *n =* 2 treatment partners, *n =* 3 participants) (Table 2, Supplemental Materials). There were no refusals or dropouts for qualitative interviews.

### Data collection

#### Implementation science framework

The Consolidated Framework for Implementation Research (CFIR) was used to guide quantitative and qualitative data collection. The updated CFIR, published after data collection materials were developed, was used for analysis [20]. The study team selected the updated CFIR constructs of ‘Tailoring Strategies’ and ‘Adapting’ from the updated CFIR as these were deemed relevant to understanding how the determinants of the implementation of the intervention were used to tailor and adapt the intervention prior to the pilot study (Table 3, Supplemental Materials) [21].

##### Data tools and collection

Facility readiness assessments were adapted from the World Health Organization package of essential noncommunicable disease interventions (WHO PEN) tool [22, 23] and included data on the number of PLWH and hypertension seen at each clinic, staff responsibilities in hypertension management, and availability of clinic resources (e.g. trained staff, guidelines, equipment) (Table 1). Four trained research assistants (described in detail below) completed the facility assessments at each study site using observation, clinic logbooks, and pharmacy stock review.

**Table 1:** Facility assessment of two HIV study clinics

Assessment	L clinic (June 2021)	S clinic (November 2020)
HIV and hypertension participants/month	700	94
blood pressure machine	Present, not working	2 functioning blood pressure machines
Computer in facility (with EHR capability)	9 total, 5 in HIV clinic all functional	8 total, 4 in HIV clinic, all functional
Electricity	Depends on national grid, no back-up generator	Depends on national grid, no back-up generator
Trained staff for hypertension management	1 family nurse practitioner (FNP)	1 FNP in the outpatient department and none in HIV
Trained staff for blood pressure measurement	No	Yes
Guidelines available	No	Botswana HIV treatment guide available, no primary care guidelines
hypertension algorithm posted	No	Yes
hypertension-related documentation in chart	None	Yes
hypertension medications on site	None at HIV clinic	Available at HIV clinic pharmacy
hypertension meds stockout last 3 months	None	None
hypertension targeted outreach or QI	None	None
Participant education materials	No	Yes
Participant flow	^a^	Well defined
Blood pressure measurement	Outpatient department	HIV clinics
hypertension management	^a^	HIV clinics and local clinics
Prescription pickup	^a^	HIV pharmacy and local clinics
Participant counseling	No	Done by nurses
Facility hypertension management	Yes	Yes, but most participants are managed at their local clinics
Initiation	Doctor	Doctor/FNP and HIV clinic nurses
Follow-up	Nurse/ Doctor	Nurses/FNP and Doctor

*FNP* family nurse practitioner, *blood pressure* blood pressure

^a^not available data

Stakeholder surveys included socio-demographics, experiences with hypertension, and attitudes towards InterCARE (1 = strongly agree, 5 = strongly disagree). For HCPs and treatment partners, surveys also included items adapted from an existing questionnaire previously used in Botswana [24] regarding confidence in managing hypertension for HCPs and HIV for treatment partners (1 = very confident, 5 = not very confident).

Semi-structured KIIs were developed guided by CFIR and other factors (e.g., HIV stigma) known to affect HIV care from prior research. KIIs were intended to explore stakeholders’ understanding of and experiences with hypertension, challenges managing hypertension in the current system, and perceptions of the InterCARE intervention (acceptability, feasibility, and relative advantage) (Table 4, Supplemental Materials).

##### Survey and KII administration

Four university-educated research assistants (3 females, 1 male) fluent in both the local language, Setswana, and in English underwent survey administration and qualitative research training. These trained research assistants pilot tested surveys and KIIs on all key stakeholder groups for feasibility and readability. Informed consent of eligible stakeholders was collected prior to survey and KII administration. Surveys and KIIs were conducted anonymously by the same research assistants who had no prior relationships with the participants. Surveys and KIIs were conducted in English and/or Setswana, based on participant preference, for approximately 30 min in a private room at the clinic. Aside from the interviewee and interviewer, no other individuals were present in the room. KIIs were audio-recorded, transcribed, and translated to English where necessary. No field notes were made during the interviews. A native Setswana speaker (KS) from the study team independently verified the translations. No repeat interviews were carried out, and no transcripts were returned to the participants for comment or correction. A set number of interviews were planned based on time and resources, with no protocol to collect data until data saturation.

#### Study population

All PLWH and hypertension eligible and enrolled in the pilot phase of InterCARE underwent a baseline study visit with trained research assistants that included measurement of anthropometric data and collection of self-reported socio-demographic, economic, and clinical data. Three left arm blood pressure readings using an automated blood pressure cuff were taken during the initial study visit [25]. An average of the three blood pressure readings, weights, and height measurements were used in analysis. Participant health records were accessed by research assistants to collect co-morbidity, prescription, and laboratory data for enrolled participants.

### Data analysis

#### Quantitative

Anthropometric, socio-demographic, and clinical (co-morbidities, laboratory data) data were summarized using descriptive statistics. Clinical data were used to calculate the WHO CVD Risk Score. As lipids were not readily available for most participants, the non-laboratory based risk charts were primarily used [26]. Uncontrolled blood pressure cut-offs were selected based on 2016 Botswana National Primary Care guidelines, defined as a systolic blood pressure of ≥ 140 or diastolic blood pressure ≥ 90 mm Hg in non-diabetic participants and a systolic blood pressure of ≥ 130 or diastolic blood pressure ≥ 80 mm Hg in participants with diabetes [27]. Chi-square, *t* test, and Wilcoxon’s rank sum statistics were calculated to compare participant characteristics between those with uncontrolled blood pressure and those with controlled blood pressure.

Descriptive statistics were used to summarize survey and facility assessment data. Based on the distribution of data, survey responses were re-categorized from Likert scales into a binary variable. Strongly agree or very confident (1) and agree or confident (2) were categorized as “agree” and “confident”, respectively. All other responses (3–5) were re-coded as “does not agree” or “not confident”, respectively. Data were organized by CFIR constructs, and each construct was coded as a facilitator ( +), barrier (-), or both facilitator and barrier ( ±). All analyses were completed in Stata Statistical Software (17.0; College Station, 2021).

#### Qualitative

KIIs were transcribed, and direct deductive content analysis guided by CFIR was completed [28]. Two investigators (PG, NY) read all of the transcripts and independently manually coded the same two full transcripts in Microsoft Word to identify preliminary codes. After discussion of these codes and use of consensus strategies to resolve disagreements, an initial codebook was created and applied to transcripts. In subsequent meetings, a final codebook was agreed upon, and subthemes were expanded and mapped onto the updated CFIR domains and constructs [29]. Coding and subthemes were reviewed by an additional investigator, and both investigators met with senior investigators to reach consensus. Participants did not provide feedback on the findings.

#### Mixed method analysis

A convergent parallel design was used [30, 31]. The study was designed, and data were collected and analyzed according to best practices commissioned by the Office of Behavioural and Social Sciences Research at the NIH and written by Creswell JW et al. Quantitative and qualitative data were collected simultaneously and analysed independently (Table 4, Supplemental Materials). The results were compared to identify areas of convergence and divergence. Findings were discussed with the research team to validate the results [32].

#### Adherence to reporting guidelines

The COREQ checklist report was used to report qualitative methods and results in this manuscript. 

#### Patient and public involvement

Patients and key stakeholders were engaged when designing survey tools and KIIs. Study findings were disseminated to local and Ministry of Health (MOH) clinical staff and key stakeholders.

## Results

### Gaps in current care

Baseline data were collected on a total of 290 PLWH and hypertension (22.8% male, mean age 54 (SD 11) at the study clinics. HIV viral load was suppressed (< 400 copies/ul) in 97.5 of the population, and 72.5% had a last CD4 count above 500 cells/ul. Obesity (BMI >  = 30kg/m^2^) was present in 25.5% of the population. Most (90.3%) participants reported taking medications for hypertension, but only 47.2% had controlled blood pressure. The mean systolic blood pressure was 135 mmHg (SD 18 mmHg), and the mean diastolic blood pressure was 88 mmHg (SD 13 mmHg). Male gender (*p* < 0.01) and decreased CD4 count (*p* = 0.04) were significantly associated with uncontrolled blood pressure. There were no significant associations of blood pressure control with age, education, employment status, household income, viral load, and BMI (Table 2).

**Table 2:** Baseline Characteristics of Baseline Study Population

Characteristic	Controlled blood pressure	Uncontrolled blood pressure	Total	*p*-value
**N**	137 (47.2%)	153 (52.8%)	290 (100.0%)	
**Gender**				
Male	19 (13.9%)	47 (30.7%)	66 (22.8%)	** < 0.01**
**Mean age (SD)**	54.3 (10.9)	53.6 (11.3)	53.9 (11)	0.60
**Highest education**				
Less than primary school	24 (17.5%)	38 (24.8%)	62 (21.5%)	0.16
Primary school	63 (46.0%)	56 (36.6%)	119 (41.3%)	
Secondary school	41 (29.9%)	54 (35.3%)	95 (32.8%)	
Higher than secondary	9 (6.6%)	5 (6.6%)	14 (4.5%)	
**Employment status**				
Employed	47 (34.3%)	57 (37.3%)	104 (35.9%)	0.60
Unemployed	90 (65.7%)	96 (62.7%)	186 (64.1%)	
**Monthly household income in Pula**^a^				
No income	37 (27.0%)	29 (19.0%)	66 (22.8%)	0.53
< 500	24 (17.5%)	39 (25.5%)	63 (21.7%)	
500–999	36 (26.3%)	40 (26.1%)	76 (26.2%)	
1000 – 4,999	32 (23.4%)	35 (22.9%)	67 (23.1%)	
≥ 5000	3 (2.2%)	6 (3.9%)	9 (3.1%)	
Do not want to answer/do not know	5 (3.6%)	4 (2.6%)	9 (3.1%)	
**Clinic location**				
S Clinic	29 (21.2%)	21 (13.7%)	50 (17.2%)	0.09
L Clinic	108 (78.8%)	132 (86.3%)	240 (82.8%)	
**Most recent CD4 count in past two years**				
CD4 less than 200	3 (0.8%)	1 (0.6%)	4 (1.6%)	**0.04**
CD4 from 200–499	23 (16.8%)	46 (30.1%)	69 (26.0%)	
CD4 at or above 500	97 (70.8%)	95 (62.1%)	192 (72.5%)	
**Percentage with undetectable viral load (VL < 400)**	126 (96.9%)	146 (98.0%)	272 (97.5%)	0.57
**WHO CVD Risk** [26]				0.63
< 10%	84 (94.4%)	105 (90.5%)	189 (92.2%)	
10 to < 20%	3 (3.4%)	4 (3.5%)	7 (3.4%)	
20 to < 40%	0	0	0	
40 + %	1 (1.1%)	3 (2.6%)	4 (2.0%)	
Smoking (current)	9 (6.6%)	16 (10.5%)	25 (8.6%)	0.16
**Percentage with documented diabetes in HIV health record**	2 (1.5%)	1 (0.01%)	3 (1.0%)	–-
**Body Mass Index**				
Underweight	8 (5.8%)	12 (7.8%)	20 (6.9%)	0.11
Normal weight	43 (31.4%)	58 (37.9%)	101 (34.8%)	
Overweight	42 (30.7%)	53 (34.6%)	95 (32.8%)	
Obese	44 (32.1%)	30 (19.6%)	74 (25.5%)	

*BP* blood pressure, *CD4* helper T lymphocyte cells, *CVD* cardiovascular disease, *SD* standard deviation, *VL* viral load, *WHO* World Health Organization

^a^1 Pula to 0.07 USD

### Determinants of implementation by CFIR domains

#### CFIR innovation domain

##### Innovation relative advantage (+)

HCPs (85%) agreed that InterCARE would be more effective than current models of care at controlling hypertension in PLWH. In KIIs, HCPs, PLWH and hypertension, and treatment partners also believed that InterCARE would be advantageous in reducing transportation costs for PLWH and hypertension, improving medication adherence, and improving patient education compared to the current model of care (Table 3; Table 5, Supplemental Materials).

*"Because there is a combined screening and consultation for HIV and cardiovascular diseases… [there is a reduced] wait time, [reduced] number of visits to the clinic…and patients are [equipped] with knowledge [to manage their hypertension] in regards to diet and exercise.”*

-Nurse

**Table 3:** InterCARE facilitators and barriers identified using CFIR constructs

Summary	Quantitative data	Qualitative data
**CFIR innovation domain**		
**Innovation relative advantage ( +)**		
-InterCARE will be more effective than current models -Reduction in clinic visits, transportation costs, improved medication adherence and participant education	-85% HCPs agreed InterCARE more effective than current models of care	*"Because there is a combined screening and consultation for HIV and cardiovascular diseases… [for patients there is a reduced] wait time, [reduced] number of visits to the clinic…and patients are [equipped] with knowledge [to manage their hypertension] in regards to diet and exercise.” -Nurse*
**Innovation adaptability (+/-):**		
-Concern about intervention adaptability, but may not require much adaptation given baseline clinic services	-35% HCPs agreed InterCARE would be difficult to adapt to meet patient needs	*“You already have [the services] in place [independently] and now [they would be integrated], so I don’t think [it would be difficult] to maneuver around [services] and achieve integration.” -Nurse*
**Innovation complexity (-)**		
-Mixed feeling amongst groups regarding complexity of integrated care	-25% of HCPs and 30% of community members felt InterCARE (combining hypertension and HIV care into the same clinic visit) -38% of community members and 40% treatment partners felt that using TPs to manage both HIV and hypertension would be too complicated	*No associated qualitative data*
**Innovation design ( +)**		
-Overall positive perception of design of InterCARE with belief that it would be impactful	-80% of TPs, over 85% of HCPs and 98% of community members believed InterCARE would be successful, would be easy to understand and have a positive impact	*“…all services must be done in same room (place) without a person moving from one place to another. Also, by giving medications at same place [for HIV and hypertension] without [telling us to] take AR[T] from one side and take high blood pressure medications from the other side, you have come with a good program by combining all of these services.” -PLWH and hypertension*
**CFIR inner setting domain**		
**Structural characteristics (+/-)**		
-Staff shortages at baseline, mixed feelings regarding burden of work -Nurses typically led new initiatives -eadership structure already in place to support implementation	-65% of HCPs agreed too much staff was required for InterCARE -40% of HCPs agreed that there were not enough supportive care resources for integrated care Doctors initiate and manage hypertension in L clinic, but not S clinic	*“We [as HCPs] deal with patients having to come for a certain service, and tomorrow they are coming for a different service…but knowing that [a patient] might come here once and still be able to get help for 2 or 3 ailments [could prevent staff from overworking and allow participants to utilize their clinic time more efficiently].” -Public health officer* *"We don’t have a medical doctor in the cluster so I would say nurses are the ones who take the lead for the new initiatives of improving the care" -Nurse* *"We have leaders like district leaders, matrons, chief doctors, also [there] are cluster matrons and facility matrons…They will be able to take the rightful administrative steps…in order for the program to run smoothly."* –*Nurse*
**Compatibility ( +)**		
-Compatible with needs of PLWH and hypertension -Familiarity with treatment partner strategy	90% of HCPs agreed intervention was compatible with needs of PLWH and hypertension ≥ 95% treatment partners and community members agree that treatment partners could successfully help PLWH and hypertension	*“For instance, if somebody has been involved in a road traffic accident and has a fracture… [relatives or close partners take care of that patient so a treatment partner is used for many conditions].* – *Nurse* *“I do not have any challenges since my treatment partner gives me full support towards my diet, what to/what not to do.”-PLWH and hypertension*
**Available resources (-)**		
- Missing or non-functioning blood pressure cuffs -Electricity cuts with no backup generators -Medication stock outs reported by patients but not clinics	Non-functioning equipment, gaps in electricity 40% HCPs felt that there were inadequate medical and supportive care resources to manage PLWH and hypertension Neither clinic reported medication stock outs in the past 3 months	*"We having been reporting that we having defunct bathroom scale…[and] we work with only one [blood pressure] monitor, the other one is not working…the[re] is a regular cut of electricity [and] since our blood pressure machines rely on electricity it means that the checking of blood pressure and pulse rate may be compromised.” -Nurse* *“…sometimes you will go to a clinic where the medication is out of stock, and you end up going to X where the medication will also be out of stock…Other challenges could be… travelling a long distance to come for [a] medication refill.”- PLWH and hypertension*
**Access to knowledge and information (-)**		
-Limited access to guidelines and continued professional development -HCPs requesting additional training	National hypertension or primary care guidelines not available in either clinic, hypertension algorithm available in S clinic only 15% of HCPs received hypertension training in past two years	*" I believe [there can be a] large scale training for dispenser[s] and nurses in the clinic. Maybe you can lobby for it or recommend [it]." -Nurse*
**CFIR individuals domain**		
**Innovation deliverers (-)**		
-HCPs confidence in hypertension medication management was lacking -TPs requested additional support (both from staff and other TPs) to manage both hypertension and HIV	HCPs felt confident diagnosing hypertension but less confident prescribing and adjusting hypertension medications TPs confident in HIV management, concerned about increased workload managing HIV and hypertension and requesting additional support	*No associated qualitative data*
**Opinion leaders ( +)**		
-Involvement of local leaders recommended to promote intervention	No associated quantitative data	*" The Kgosi [chiefs] are the gate keepers to the village so if they are receptive of initiative, chances are people are going to be [accepting]…”- Public health officer*
**Implementation facilitators ( +)**		
-Model PLWH and hypertension patients (champions) could act as natural advocates for the intervention	No associated quantitative data	*"They [model patients] are in a position to share their personal experiences [and positively influence their peers]. [Their peers] might be paranoid [about this new thing, but will join if they see that other members in the community are participating]. -Nurse*
**CFIR outer setting domain**		
**Local attitudes (+/-)**		
-Mixed experiences with HIV stigma, no hypertension stigma reported	No associated quantitative data	*“Yes, stigma is very common… Even at my household there is so much stigma, I always hear [my family] criticising people living with HIV [which] is why I opted to open up only to my child because I wanted to avoid their stressful comment[s]”- Treatment partner* *“No sir there is no stigma [against high blood pressure]…this thing is now common [and is the] same as taking ARV or taking any pills…You see when AIDS started [there was stigma]. These days a person can go in public saying “I’m going to charge meaning ARV’’ -PLWH and hypertension*
**Updated CFIR implementation process domain**^**a**^		
**Tailoring strategies**		
-Treatment partner strategy: training was expanded to include support from HCPs and research staff, and training videos		
**Adapting**		
-Task sharing strategy: hypertension management tasks split amongst nurses, FNPs, and doctors. All HCPs were supported and coached by the study nurse and study physician -Community and clinic engagement: local leadership was actively involved in supporting and improving the intervention both by providing suggestions for improvement and working with the study team to ensure necessary resources and training would be made available		

^a^A summary of tailoring and adapting that occurred by the study team after data collected was analyzed

##### Innovation adaptability (+/-)

In surveys, over a third of HCPs agreed that it would be difficult to adapt InterCARE to meet the needs of PLWH and hypertension. In contrast, HCPs interviewed in KIIs felt it would not be difficult to adapt InterCARE to meet the needs of PLWH with hypertension since many services for both conditions were already available.

*“You already have [the services] in place [independently] and now [they would be integrated], so I don’t think [it would be difficult] to maneuver around [services] and achieve integration.”*

-Nurse

##### Innovation complexity (-)

In survey data, 25% of HCPs and 30% of community members believed that implementing InterCARE in the clinic would be too complex. Regarding the use of treatment partners, 38% of community members and 40% of treatment partners report that it would be too complicated to have treatment partners manage both HIV and hypertension. There were no associated qualitative data collected.

##### Innovation design (+)

In surveys, treatment partners (80%), community members (98%), and HCPs (90%) thought that InterCARE would be successful at improving the treatment of hypertension for PLWH and hypertension. HCPs (85%) also agreed that the intervention would be easy to understand (85%) and would benefit PLWH and hypertension (95%). The KIIs also reflected these results with a positive perception of integration of HIV and hypertension services by all groups interviewed, including PLWH and hypertension.

*“…all services must be done in same room (place) without a person moving from one place to another. Also, by giving medications at same place [for HIV and hypertension] without [telling us to] take AR[T] from one side and take high blood pressure medications from the other side, you have come with a good program by combining all of these services.”*

-PLWH and hypertension

### CFIR inner setting domain

#### Structural characteristics—work infrastructure (-/ +)

Perspectives on staffing needs prior to implementing InterCARE were variable. In surveys, two-thirds of HCPs (65%) thought InterCARE would require too many staff and other resources.

In KIIs, divergent from survey data HCPs noted in the long-term consolidating clinic visits would decrease the clinical care load.

*“We [as HCPs] deal with patients having to come for a certain service, and tomorrow they are coming for a different service…but knowing that [a patient] might come here once and still be able to get help for 2 or 3 ailments [could prevent staff from overworking and allow participants to utilize their clinic time more efficiently].”*

-Public health officer

The facility readiness assessment identified the different models between clinics, with doctors predominantly initiating and managing hypertension in L clinic compared to more task sharing amongst nurses and doctors in the smaller, more remote S clinic (Table 1).

In KIIs, respondents noted that nurses typically lead new initiatives, a significant factor for successful implementation of InterCARE. A doctor was not always readily available in person, particularly at smaller clinics.

*"We don’t have a medical doctor in the cluster so I would say nurses are the ones who take the lead for the new initiatives of improving the care"*

-Nurse

HCPs discussed that the leadership structure within the clinic could serve as a facilitator to support implementation of InterCARE.

*"We have leaders like district leaders, matrons, chief doctors, also [there] are cluster matrons and facility matrons…They will be able to take the rightful administrative steps…in order for the program to run smoothly."*

-Nurse

#### Compatibility (+)

In surveys, 90% of HCPs agreed that InterCARE was compatible with the needs of PLWH and hypertension at clinic. Nearly all treatment partners and community members agreed that treatment partners could successfully help participants manage their hypertension.

In KIIs, it was noted that the use of treatment partners for hypertension is also consistent with the current HIV treatment partner program. In addition, outside of HIV, family and friends informally fill the role of treatment partners for acute and chronic conditions. PLWH and hypertension had a positive view of treatment partners, describing their multi-faceted role in medication and clinic appointment reminders, disease counselling and emotional support.

*“For instance, if somebody has been involved in a road traffic accident and has a fracture… [relatives or close partners take care of that patient so a treatment partner is used for many conditions].*

–Nurse

*“I do not have any challenges since my treatment partner gives me full support towards my diet, what to/what not to do.”*

-PLWH and hypertension

#### Available resources (-)

Resource limitations in equipment were documented in the facility readiness assessment, including dysfunctional blood pressure machines and no hypertension medications at L clinic. In surveys, 40% of HCPs agreed that InterCARE would be problematic due to not having enough medical and supportive care resources to care for both HIV and hypertension. KIIs confirmed the lack of essential equipment at baseline (electricity, blood pressure cuffs, working scales) and re-iterated the importance of having available resources to implement InterCARE.

*“The[re] is a regular cut of electricity [and] since our blood pressure machines rely on electricity it means that the checking of blood pressure and pulse rate may be compromised.”*

-Nurse

In KIIs, stakeholders discussed medication stock-outs and long distances to the clinic, both contributing to defaulting on medications.

*“…sometimes you will go to a clinic where the medication is out of stock, and you end up going to X where the medication will also be out of stock…Other challenges could be…travelling a long distance to come for [a] medication refill.”*

-PLWH and hypertension

#### Access to knowledge and information (-)

In the facility readiness assessment, prior HCP training on hypertension care, and availability of national hypertension guidelines and patient education materials were variable between the two clinics. In surveys, only three (15%) HCPs reporting receiving hypertension training in the past two years. In KIIs, HCPs requested more dedicated hypertension training.

*"I believe there can be a large-scale training for dispensers and nurses in the clinic, maybe you can lobby for it or recommend for it."*

-Nurse

### CFIR individuals domain

#### Innovation deliverers (-)

HCPs responsible for direct patient care (nurses, FNPs, doctors) had differing levels of confidence with different components of hypertension management. HCPs felt confident counseling patients on diet for hypertension and identifying uncontrolled hypertension. Few felt confident prescribing medications for hypertension (36.4%) or adjusting medications when hypertension is not controlled (27.3%).

Most treatment partners (70%) agreed to having the confidence and knowledge to help patients manage HIV. Over half (60%) felt they were being expected to do too many things as a treatment partner for HIV, and most (90%) reported needing additional support to complete their job as a treatment partner for HIV (Table 6, Supplemental Materials). There were no associated qualitative data collected.

#### Opinion leaders ( +)

In KIIs, there was an emphasis on the importance of communicating the intervention in public forums (e.g., kgotla – tribal council) through trusted community members (e.g., nurses and Kgosi – chief) to ensure public acceptance.

*"The Kgosi [chiefs] are the gate keepers to the village so if they are receptive of [the] initiative, chances are people are going to be [accepting].”*

-Public health officer

#### Implementation* facilitators *( +)

*In KIIs,* champions or model patients (with HIV or hypertension) were identified as natural advocates who could promote the intervention, medication adherence, and health lifestyle behaviors in the community.

*"They [model patients] are in a position to share their personal experiences [and positively influence their peers]. [Their peers] might be paranoid [about this new thing, but will join if they see that other members in the community are participating].*

-Nurse

### CFIR outer setting domain

#### *Local attitudes* (+/-)

HIV and hypertension related stigma were discussed in KIIs. For some, HIV stigma was seen as a major barrier to care. Certain clinic structures did not allow participants with HIV to remain anonymous, and some PLWH and treatment partners were unwilling to disclose their HIV status to peers. Others reported more acceptance of HIV. No stakeholder reported any hypertension related stigma when prompted.

*“Yes, stigma is very common... Even at my household there is so much stigma, I always hear [my family] criticising people living with HIV…”*

-Treatment partner

*“No sir there is no stigma [against high blood pressure]…this thing is now common [and is the] same as taking ARV or taking any pills…You see when AIDS started [there was stigma]. These days a person can go in public saying “I’m going to charge meaning AR[T]’’*

-PLWH and hypertension

Additional qualitative themes and associated quotations are summarized in Table 7, Supplemental Materials.

### CFIR implementation process domain

Prior to implementation and at the start of the pilot study, the mixed methods results were reviewed and discussed by the study team. These results supported existing InterCARE intervention strategies and guided tailoring and adapting prior to the pilot study (Table 4). The updated CFIR implementation process domain was used to characterize these tailored strategies and adaptations.

**Table 4:** InterCARE intervention original strategies and adaptations

Strategy	Description
Electronic Health Records (EHR)	*• Provider prompts:* record blood pressure and weight, screen and counsel about physical activity, low salt diet, and weight management, screen and counsel on safe alcohol consumption and smoking cessation, and screening and recording of CVD risk factors *• Provider automatic reminders:* diagnosis and action for any out-of-range blood pressure value (> 140/90 mmHg or > 130/80 mmHg if they have underlying diabetes mellitus or chronic kidney disease) *• Documentation:* blood pressure, body mass index, CVD risk factors (kidney function, diabetes screening, cholesterol screening), hypertension medications prescribed, hypertension and risk factor counselling performed
Healthcare provider training	*•* Online course of ten modules with pre- and post-test assessments *•* One-on-one coaching **Adaptations** *• Task sharing strategy*: split hypertension management tasks amongst nurses, FNPs, and doctors ◦ Nurses: vitals, participant education, follow-up care, CVD screening ◦ FNPs and doctors: medication initiation, complex hypertension care
Treatment partners	*•* Pair participants with self-selected treatment partner *• Role of treatment partner:* communicate at least weekly with participant to provide counselling and support on medication adherence and attending clinic appointments, hypertension knowledge, and healthy diet and exercise **Tailoring:** *•* Training videos *•* Support and one-on-one instruction from healthcare providers
Engagement and Liaison with Ministry of Health, Clinic Leadership, Community Leadership and Key Stakeholders	*•* Prior to the pilot study, senior staff members met with: ◦ The Ministry of Health NCD Committee to review the intervention, provider training and distribution of guidelines in participating clinics ◦ Chief and local leadership regarding sensitizing the community to the intervention ◦ Local District Health Management Teams to discuss the intervention and obtain letters of support to distribute to participating clinics and strategize regarding supplying clinics with necessary resources (e.g., blood pressure cuffs) ◦ Clinic leadership to discuss and adapt the intervention and plan implementation together

#### Tailoring strategies

To improve the treatment partner strategy and bridge gaps in access to knowledge and information, efforts were made to expand training provided to treatment partners. In addition to the existing one-on-one instruction and support from HCPs and research staff, training videos were created by the study staff specifically for treatment partners.

#### Adapting

To strengthen the provider training strategy and address staffing shortages reported, the intervention was adapted and a task sharing strategy was added by the study team. Task sharing procedures to split hypertension management tasks amongst nurses (e.g., vitals, participant education, follow-up care, CVD screening) and FNPs and doctors (e.g., medication initiation, complex hypertension care) were included in training. HCPs were also supported and coached by the study nurse and study physician to improve confidence in managing hypertension, particularly prescribing and adjusting medications.

Outside of the three core components of InterCARE, other strategies that were identified as crucial for successful implementation included engaging and partnering with community leaders, clinic leadership, and key stakeholders. Senior members of the study staff met with the chiefs and local leadership to discuss the study to gain support and suggestions to ensure effective implementation prior to commencing the pilot study. To address available resources, meetings between senior study staff and clinic leadership and engagement of the MOH were necessary to ensure that electricity, working equipment, and medications were available.

Not all facilitators were used to tailor and adapt strategies and not all barriers were addressed by the CFIR implementation process domain. For example, HCPs identified model patients as facilitators to mobilize community participation. However due to possible stigma towards having HIV and HTN expressed by PLWH and HTN, the team chose not to include any adaptations that incorporated the use of model patients.

## Discussion

In this study population in Botswana, we found that while nearly all of the PLWH and hypertension enrolled were already on antihypertensive treatment, over half had uncontrolled blood pressure. Integrated HIV and hypertension care as implemented through InterCARE was perceived to be an advantageous, compatible intervention design by participants, HCPs, treatment partners, and community members to address the gap in effective delivery of hypertension treatment and blood pressure control amongst PLWH. Significant barriers to implementation of InterCARE arose in the CFIR inner setting constructs of available resources, structural infrastructure (e.g., levels of staffing), and access to knowledge and information which informed the adaptations for InterCARE implementation strategies prior to the pilot study.

The intervention design was viewed favorably, particularly the treatment partner component. Stakeholders expressed their familiarity with treatment partners and recognized its compatibility with the existing HIV clinic infrastructure in Botswana. Recognizing the complex task of integrating hypertension and HIV care, most studies including InterCARE use multiple implementation strategies to achieve integration [17, 33, 34]. The three components of the InterCARE intervention (treatment partners, modified EHR, provider training), particularly the treatment partner component, were chosen carefully and specifically for Botswana based on the strategies that had been successful in controlling the HIV epidemic in Botswana, and to optimize existing clinic structures [5, 7–9].

Identified barriers to intervention implementation included available resources and insufficient staffing for the workload required. These findings are consistent with other studies conducted in Africa [29, 35]. In a study conducted at three HIV clinics of varying hypertension care cascade performance in Uganda, major barriers to hypertension and HIV integrated care included lack of available resources (e.g., functional blood pressure machines), an inadequate supply of antihypertensive medications, and concern regarding extra workload to HCPs [29]. For our pilot study, we incorporated task shifting strategies in our provider training to more efficiently use existing staff and better adapt to existing clinic work infrastructure without increasing workload. This evidence-based approach leveraging existing human resources has also been used in other African settings to address staff shortages [35–37].

Another significant barrier to intervention implementation was limited access to knowledge and information for HCPs. The importance of provider training and limited opportunities for continued professional development opportunities is a thread across many studies integrating HIV and hypertension services [35]. In a mixed-methods study of the implementation of a task-strengthening strategy for hypertension and HIV control, clinic HCPs from 29 HIV clinics in Lagos, Nigeria noted that a sub-optimal number of clinics (52%) reported access to hypertension training materials. Training was desired as long as it did not overburden health care HCPs [36]. This study had consistent findings highlighting the need for dedicated provider training in HIV/hypertension integration interventions [18, 38–40], which is a key component of the InterCARE intervention. In addition to providing continuing professional development (CPD) opportunities through provider training, one-on-one coaching and supportive supervision were added evidence-based strategies [41–43], aimed at addressing low confidence amongst HCPs.

A strength of this study design was the use of the updated CFIR to identify determinants of implementation and guide adaptations of implementation strategies. This is one of the few studies to use the updated CFIR to assess factors that influence hypertension and HIV care integration [29, 36]. This study demonstrates the utility of the updated CFIR as a guiding framework for systematic implementation of integrated care in settings outside of Botswana [20, 44]. CFIR has been used less frequently in LMICs compared to high income countries (HICs) and there is a growing amount of literature on CFIR expansions and modifications that better fit the global context. Means AR, et al. adds CFIR domains and constructs that address scalability and sustainability of an intervention and acknowledge relationships between CFIR domains and constructs [45]. This is particularly valuable in settings like Botswana where resource limitations (e.g., internet connectivity, electricity, medication availability) that are barriers to implementation of the intervention will likely also be barriers to scaling and sustaining the intervention. Addressing these limitations will likely require complementary strategies across multiple CFIR domains.

Another strength of this study was the addition of the community stakeholder perspective. Community acceptance plays a central role in health care provision in Botswana [46, 47]. Local leaders and clinic staff are important facilitators in sensitizing the population to, and promoting, integrated care. The central role that chiefs and the kgotla, the community council, play in the lives of the citizens of Botswana has long been recognized in National MOH Guidelines in Botswana as a key facilitator in healthcare delivery [46]. The addition of a strategy to engage and partner with key stakeholders, including the MOH, clinic leadership, and community leadership can serve to strengthen the delivery of InterCARE.

Limitations of this study design include the small sample size and limited perspectives in KIIs from HCPs who were prescribers of antiretroviral therapy and antihypertensives (doctors and FNPs). Time and resource limitations prevented the collection of more KIIs for all groups, which would have helped achieve data saturation and a more comprehensive assessment of barriers and facilitators to each of the three major strategies. Another limitation was a potential selection bias as all stakeholders, including community members, were recruited from the HIV clinic setting. In future studies, community recruitment may be helpful in gathering a more universal sample of opinions. Our qualitative sample size may have also contributed to divergent quantitative and qualitative data for a few CFIR constructs. As a pilot study, for some CFIR constructs saturation of themes may not have been achieved to reflect the full diversity of convergent and divergent opinions. For example, for the CFIR innovation domain construct of adaptability, one third of participants agreed that InterCARE would be difficult to adapt but the remainder disagreed with this statement. It is possible that the sampling of participants for KIIs was biased and only reflected the opinions of those that disagreed with this statement.

For other constructs, divergent data may have arisen due to differences in how participants understood the survey questions versus KII questions. However, because we used a convergent parallel mixed methods study design, in which qualitative and quantitative data were conducted during the same study phase, we were not able to use the qualitative data to help explain quantitative data (as would have been possible with an explanatory sequential mixed methods design.” [30].

Finally, this study only includes perceived determinants of implementation based on a description of the planned implementation of InterCARE. Further insights will be obtained during the two-stage type 2 hybrid effectiveness-implementation trial.

While this study focused on key facilitators and barriers that were addressed directly, further research is needed on the potential indirect effects of integrated care. In KIIs, stakeholders discussed the role of HIV stigma in care seeking behaviors. It is possible that receiving hypertension care in HIV specific clinics may have the potential to exacerbate HIV stigma affecting both HIV and hypertension care. Adding hypertension services to HIV clinics may not adequately address HIV-related stigma and will be an important factor to explore further during the pilot and randomized controlled study [48].

Botswana, like many other LMICs, is at a crossroads moving forward from the devastation of rampant, poorly controlled HIV decades earlier. Now faced with a growing epidemic of hypertension, immediate action is needed to better control hypertension in this setting. Utilizing existing HIV infrastructure through integration of services is an intervention strategy that is gaining traction worldwide [18, 49]. Our study found that the integration of hypertension and HIV services through the InterCARE intervention was viewed positively. Implementation strategies of key stakeholder engagement and partnership, provider supervised support and coaching, and task sharing can be used to address major barriers, utilize facilitators, and strengthen the existing components of InterCARE. These strategies along with the core InterCARE strategies are now being tested in a nation-wide two-stage type 2 hybrid effectiveness-implementation cluster randomized control trial.

## Conclusion

Integrating hypertension services in HIV clinics is a feasible and acceptable intervention that is envisioned to be effective in better controlling blood pressure, with potential advantages over the current standard of care. Barriers exist, but strategies to address these can be successfully adapted and will be tested in the planned pilot study.

### Supplementary Information

Supplementary Material 1.

Supplementary Material 2.

## Data Availability

This study is in compliance with the NIH Public Access Policy, which ensures that the public has access to the published results of NIH funded research. All results have been (and will be made) available from final peer-reviewed journal manuscripts (including this one) via the digital archive PubMed Central upon acceptance for publication.

## Abbreviations

PLWH: People living with HIV
CVD: Cardiovascular diseases
IDCC: Infectious Diseases Care Clinic
BCPP: Botswana HIV Combination Prevention Project
REDCap: Research Electronic Data Capture
EHR: Electronic health records
CFIR: Consolidated Framework for Implementation Research

## References

[1] Bachanas P, Alwano MG, Lebelonyane R (2021). Finding, treating and retaining persons with HIV in a high HIV prevalence and high treatment coverage country: results from the Botswana Combination Prevention Project. PLoS ONE.

[2] BS M. Botswana: Fifth Botswana AIDS Impact Survey (BAIS V). Gaborone, Botswana: Republic of Botswana, 6 September 2022 2022. (https://www.statsbots.org.bw/sites/default/files/BAIS%20V%20Preliminary%20Report.pdf).

[3] Levi J, Raymond A, Pozniak A, Vernazza P, Kohler P, Hill A (2016). Can the UNAIDS 90–90-90 target be achieved? A systematic analysis of national HIV treatment cascades. BMJ Glob Health.

[4] Mosepele M. High prevalence of HTN in HIV-infected and HIV-uninfected adults in Botswana. Conference on Retroviruses and Opportunistic Infections (CROI) Boston 2018.

[5] Wester CW, Bussmann H, Avalos A (2005). Establishment of a public antiretroviral treatment clinic for adults in urban Botswana: lessons learned. Clin Infect Dis.

[6] Ledikwe JH, Kejelepula M, Maupo K (2013). Evaluation of a well-established task-shifting initiative: the lay counselor cadre in Botswana. PLoS ONE.

[7] Bussmann C, Rotz P, Ndwapi N (2008). Strengthening healthcare capacity through a responsive, country-specific, training standard: the KITSO AIDS training program's support of Botswana's national antiretroviral therapy rollout. Open AIDS J.

[8] Galani M HD, Tibben W, Letsholo KJ. Improving continuity of HIV/AIDS care through electronic health records in resource-limited settings: a Botswana perspective. Health Policy Technol. 2021;10(2). 10.1016/j.hlpt.2021.03.001.

[9] Bogart LM, Mosepele M, Phaladze N (2018). A social network analysis of HIV treatment partners and patient viral suppression in Botswana. J Acquir Immune Defic Syndr.

[10] Ramiah I RM. Public-private partnerships and antiretroviral drugs For HIV/AIDS: lessons from Botswana. Health Aff. 2005;24(2). 10.1377/hlthaff.24.2.545. 10.1377/hlthaff.24.2.545 15757942

[11] Hsue PY, Waters DD (2018). Time to recognize HIV infection as a major cardiovascular risk factor. Circulation.

[12] Delabays B, Cavassini M, Damas J (2022). Cardiovascular risk assessment in people living with HIV compared to the general population. Eur J Prev Cardiol.

[13] Sarfo FS, Nichols M, Singh A (2019). Characteristics of hypertension among people living with HIV in Ghana: Impact of new hypertension guideline. J Clin Hypertens (Greenwich).

[14] Xu Y, Chen X, Wang K (2017). Global prevalence of hypertension among people living with HIV: a systematic review and meta-analysis. J Am Soc Hypertens.

[15] Bigna JJ, Ndoadoumgue AL, Nansseu JR (2020). Global burden of hypertension among people living with HIV in the era of increased life expectancy: a systematic review and meta-analysis. J Hypertens.

[16] Dzudie A, Hoover D, Kim HY (2021). Hypertension among people living with HIV/AIDS in Cameroon: a cross-sectional analysis from Central Africa International Epidemiology Databases to Evaluate AIDS. PLoS ONE.

[17] Birungi J, Kivuyo S, Garrib A (2021). Integrating health services for HIV infection, diabetes and hypertension in sub-Saharan Africa: a cohort study. BMJ Open.

[18] McCombe G, Lim J, Hout MCV (2022). Integrating care for diabetes and hypertension with HIV care in sub-Saharan Africa: a scoping review. Int J Integr Care.

[19] McCombe G, Murtagh S, Lazarus JV (2021). Integrating diabetes, hypertension and HIV care in sub-Saharan Africa: a Delphi consensus study on international best practice. BMC Health Serv Res.

[20] Damschroder LJ, Reardon CM, Widerquist MAO, Lowery J (2022). The updated consolidated framework for implementation research based on user feedback. Implement Sci.

[21] Damschroder LJ, Aron DC, Keith RE, Kirsh SR, Alexander JA, Lowery JC (2009). Fostering implementation of health services research findings into practice: a consolidated framework for advancing implementation science. Implement Sci.

[22] Mutale W, Bosomprah S, Shankalala P (2018). Assessing capacity and readiness to manage NCDs in primary care setting: gaps and opportunities based on adapted WHO PEN tool in Zambia. PLoS ONE.

[23] Organization WH (2013). Implementation tools: Package of Essential Noncommunicable (PEN) disease interventions for primary health care in low-resource settings.

[24] Pooja Gala, Bhavna Seth, Veronica Moshokgo, et al. Confidence and performance of health workers in cardiovascular risk factor management in rural Botswana. Lancet Glob Health 2019;7(S13) (Abstract). 10.1016/S2214-109X(19)30098-1.

[25] Whelton PK, Carey RM, Mancia G, Kreutz R, Bundy JD, Williams B (2022). Harmonization of the American College of Cardiology/American Heart Association and European Society of Cardiology/European Society of Hypertension blood pressure/hypertension guidelines. Eur Heart J.

[26] Group CRCW (2019). World Health Organization cardiovascular disease risk charts: revised models to estimate risk in 21 global regions. Lancet Glob Health.

[27] Tsima BM, Setlhare V, Nkomazana O (2016). Developing the Botswana Primary Care Guideline: an integrated, symptom-based primary care guideline for the adult patient in a resource-limited setting. J Multidiscip Healthc.

[28] Assarroudi A, HeshmatiNabavi F, Armat MR, Ebadi A, Vaismoradi M (2018). Directed qualitative content analysis: the description and elaboration of its underpinning methods and data analysis process. J Res Nurs.

[29] Muddu M, Tusubira AK, Nakirya B (2020). Exploring barriers and facilitators to integrated hypertension-HIV management in Ugandan HIV clinics using the Consolidated Framework for Implementation Research (CFIR). Implement Sci Commun.

[30] Creswell JW, Plano Clark VL. Designing and Conducting Mixed Methods Research. 3rd ed. Thousand Oaks: SAGE; 2018.

[31] Gaglio B, Henton M, Barbeau A (2020). Methodological standards for qualitative and mixed methods patient centered outcomes research. BMJ.

[32] Palinkas LA, Aarons GA, Horwitz S, Chamberlain P, Hurlburt M, Landsverk J (2011). Mixed method designs in implementation research. Adm Policy Ment Health.

[33] Muddu M, Semitala FC, Kimera I (2022). Improved hypertension control at six months using an adapted WHO HEARTS-based implementation strategy at a large urban HIV clinic in Uganda. BMC Health Serv Res.

[34] Kwarisiima D, Atukunda M, Owaraganise A (2019). Hypertension control in integrated HIV and chronic disease clinics in Uganda in the SEARCH study. BMC Public Health.

[35] Njuguna B, Vorkoper S, Patel P (2018). Models of integration of HIV and noncommunicable disease care in sub-Saharan Africa: lessons learned and evidence gaps. AIDS.

[36] Iwelunmor J, Ezechi O, Obiezu-Umeh C (2022). Factors influencing the integration of evidence-based task-strengthening strategies for hypertension control within HIV clinics in Nigeria. Implement Sci Commun.

[37] Aifah A, Onakomaiya D, Iwelunmor J (2020). Nurses’ perceptions on implementing a task-shifting/sharing strategy for hypertension management in patients with HIV in Nigeria: a group concept mapping study. Implement Sci Commun.

[38] Muddu M, Ssinabulya I, Kigozi SP (2021). Hypertension care cascade at a large urban HIV clinic in Uganda: a mixed methods study using the Capability, Opportunity, Motivation for Behavior change (COM-B) model. Implement Sci Commun.

[39] MatanjeMwagomba BL, Ameh S, Bongomin P (2018). Opportunities and challenges for evidence-informed HIV-noncommunicable disease integrated care policies and programs: lessons from Malawi, South Africa. Swaziland and Kenya AIDS.

[40] Patel P, Speight C, Maida A (2018). Integrating HIV and hypertension management in low-resource settings: lessons from Malawi. PLoS Med.

[41] Schwerdtle P, Morphet J, Hall H (2017). A scoping review of mentorship of health personnel to improve the quality of health care in low and middle-income countries. Global Health.

[42] Arsenault C, Rowe SY, Ross-Degnan D (2022). How does the effectiveness of strategies to improve healthcare provider practices in low-income and middle-income countries change after implementation? Secondary analysis of a systematic review. BMJ Qual Saf.

[43] Iyasere CA, Baggett M, Romano J, Jena A, Mills G, Hunt DP (2016). Beyond continuing medical education: clinical coaching as a tool for ongoing professional development. Acad Med.

[44] King DK, Shoup JA, Raebel MA (2020). Planning for implementation success using RE-AIM and CFIR frameworks: a qualitative study. Front Public Health.

[45] Means AR, Kemp CG, Gwayi-Chore MC (2020). Evaluating and optimizing the consolidated framework for implementation research (CFIR) for use in low- and middle-income countries: a systematic review. Implement Sci.

[46] Ministry of Health and Wellness. National guideline for implementation of integrated community-based health services in Botswana. Gaborone: Government of Botswana; 2020.

[47] Allen THS (2004). HIV/AIDS policy in Africa: what has worked in Uganda and what has failed in Botswana?. J Int Dev.

[48] Ameh S, D'Ambruoso L, Gomez-Olive FX, Kahn K, Tollman SM, Klipstein-Grobusch K (2020). Paradox of HIV stigma in an integrated chronic disease care in rural South Africa: Viewpoints of service users and providers. PLoS ONE.

[49] Osetinsky B, Hontelez JAC, Lurie MN (2019). Epidemiological and health systems implications of evolving HIV and hypertension in South Africa and Kenya. Health Aff (Millwood).

