# Supplementary material for: A mixed methods approach identifying facilitators and barriers to guide adaptations to InterCARE strategies: an integrated HIV and hypertension care model in Botswana
Source: Implement Sci Commun. 2024 Jun 20;5:67. doi: 10.1186/s43058-024-00603-x (PMC11188218; doi:10.1186/s43058-024-00603-x)
Supplement: Supplementary file 1 — Supplementary Material 1. [file 43058_2024_603_MOESM1_ESM.docx]

*Supplemental Materials*

Table of Contents

| Reflexivity Statement | 2 |
| --- | --- |
| Table 1: Characteristics of key stakeholders | 4 |
| Table 2: Baseline characteristics of qualitative interview participants | 5 |
| Table 3: Definitions of CFIR 2.0 domains and constructs used to understand factors that may influence integrated HIV and HTN care | 6 |
| Table 4: Quantitative survey and key informant interview questions mapped by CFIR domain and construct | 7 |
| Table 5: Survey responses mapped to Consolidated Framework for Implementation Research (CFIR) constructs | 10 |
| Table 6: Confidence and Training Managing HIV and HTN | 12 |
| Table 7: Additional Qualitative Themes and Quotations | 13 |
| Key Informant Interviews |  |
| Patient | 16 |
| Treatment partners | 20 |
| Healthcare providers | 24 |
| Community members | 27 |
| Survey Tools |  |
| Patient | 30 |
| Treatment partners | 34 |
| Healthcare providers | 38 |
| Community members | 40 |

Reflexivity Statement

**Reflexivity Statement:**

This study is a collaboration between University of Botswana, Botswana Harvard Partnership and the Ministry of Health (MoH) of Botswana. In the last decade, the MoH of Botswana has made tackling non-communicable diseases and cardiovascular disease a national priority. The strategies used to integrate HIV clinics with hypertension (HTN) services were chosen in collaboration with the MoH and local leadership based on the recommendations and the needs of local communities. At the start of the national antiretroviral therapy program for HIV, Botswana developed a local electronic health record (EHR). Our team in consultation with the MoH and in conjunction with system updates already planned for the EHR system, incorporated changes to facilitate the integration of HIV and HTN services. Treatment partners have been widely adopted in Botswana for HIV and were a natural strategy already in place to also use for HTN. Finally, past work by the study team in Botswana highlighted the need and desire for provider training in HTN and cardiovascular disease.

Data were analyzed as a collaborative effort between statisticians and researchers in the United States and United Kingdom across institutions and statisticians and researchers at the University of Botswana. Meetings were convened between researchers in Botswana and colleagues in the US to interpret the study data. Co-authorship and teamwork were used as a cross-institutional capacity building partnership to support local junior researchers in developing manuscript writing skills. Through collaboration with key local stakeholders, this project contributes to improvement in the local clinic infrastructure, particularly in regards to developing a sustainable plan to ensure adequate resources for healthcare services. Safeguards used to protect local study participants and researchers include involving key stakeholders in every step of the research process, starting with obtaining permission from local clinics and local governmental leadership prior to starting any phase of the project. This project is an initiative of the local research team with research team members living abroad serving primarily as consultants and advisors.

This project was funded by the HLB SIMPLe Alliance, sponsored by the NHLBI. As a member of this alliance, the Botswana team has participated in collaborative efforts with other low-and-middle income countries (LMICs) and capacity building and training programs for junior research scientists living in LMICs.

**Table 1: Characteristics of Key Stakeholders**

| **Characteristic** | **PLWH and HTN**  **(n=20)** | **HCWs* (n=20)** | **Treatment Partners**  **(n=20)** | **Community Members+ (n=40)** |
| --- | --- | --- | --- | --- |
|  | N (%) | N (%) | N (%) | N (%) |
| **Gender** | | | | |
| Male | 10 (50.0) | 9 (45.0) | 1 (5.0) | 17 (42.5) |
| **Age** | | | | |
| 21 – 30 years | 3 (15.0) | 2 (10.0) | 4 (20.0) | 4 (10.0) |
| 31 - 40 years | 4 (20.0) | 7 (35.0) | 4 (20.0) | 17 (42.5) |
| 41 - 50 years | 5 (25.0) | 8 (40.0) | 7 (35.0) | 12 (30.0) |
| >50 years | 8 (40.0) | 3 (15.0) | 5 (25.0) | 7 (17.5) |
| **Highest Education** | | | | |
| Less than Primary School | 5 (25.0) | 0 | 0 | 0 |
| Primary and Secondary School | 14 (70.0) | 0 | 18 (90.0) | 40 (75.0) |
| Higher than Senior Secondary | 1 (5.0) | 20 (100.0) | 2 (10.0) | 10 (25.0) |
| **Employment Status (n=80)** | | | | |
| Employed | 5 (25.0) | 20 (100.0) | 10 (50.0) | 35 (87.5) |
| Unemployed | 15 (75.0) | 0 | 10 (50.0) | 5 (12.5) |
| **Monthly Household Income in Pula^ (n=80)** | | | | |
| <1000 | 9 (45.0) |  | 11 (55.0) | 9 (30.0) |
| 1001 – 4,999 | 6 (30.0) |  | 7 (35.0) | 11 (36.7) |
| ≥5000 | 1 (5.0) |  | 2 (10.0) | 10 (33.3) |
| **Clinic Location** | | | | |
| S Clinic | 10 (50.0) | 10 (50.0) | 10 (50.0) | 20 (50.0) |
| L Clinic | 10 (50.0) | 10 (50.0) | 10 (50.0) | 20 (50.0) |

*5% doctors, 5% family nurse practitioners (FNPs), 45% nurses, 10% pharmacists, 35% other (nursing assistant, health educators)

+7.5% community leader, 50% consumer services sector, 42.5% local counsel

^ 1 pula = 0.07 USD

**Table 2: Baseline Characteristics of Qualitative Interview Participants**

| **Position** | **Location** | **Age** | **Gender** |
| --- | --- | --- | --- |
| Nurse | S | 44 | Male |
| Community Member | S | 30 | Female |
| Treatment Partner | S | 54 | Female |
| Participant | S | 55 | Female |
| Community Member | S | 59 | Female |
| Public Health Officer | L | NA | Male |
| Participant | L | 66 | Male |
| Treatment Partner | L | 59 | Female |
| Participant | L | 68 | Male |
| Community Member | L | 27 | Female |

**Table 3: Definitions of CFIR 2.0 domains and constructs used to understand factors that may influence integrated HIV and HTN care**

| **CFIR Domain** | **CFIR Construct** | **Definition** |
| --- | --- | --- |
| Innovation | Relative Advantage | Is InterCARE more effective than the status quo? |
|  | Adaptability | Can InterCARE be modified to fit the local context? |
|  | Complexity | What is the scope of InterCARE and number of components required? |
|  | Design | How well is InterCARE packaged, designed and presented? |
| Inner Setting | Structural Characteristics | What is the work infrastructure at clinics, task distribution amongst staff and general staff levels? |
|  | Compatibility | How does InterCARE fit within existing systems? |
|  | Available Resources | What is the availability of resources needed to implement and deliver InterCARE? |
|  | Access to Knowledge and Information | What training is accessible to implement and deliver InterCARE? |
| Individuals | Innovation Deliverers | What is the role of individuals that directly or indirectly deliver InterCARE? |
|  | Opinion Leaders | What influence do individuals who influence the attitudes and behaviors of others have in implementing InterCARE? |
|  | Implementation Facilitators | Who are the individuals with subject matter expertise who support implementation of InterCARE? |
| Outer Setting | Local Attitudes | What are the beliefs and stigma around HIV and HTN? |

**Table 4: Quantitative Survey and Key Informant Interview Questions Mapped by CFIR Domain and Construct**

| **CFIR Domain** | **Survey** | **Key Informant Interview** |
| --- | --- | --- |
| **Innovation** | | |
| Complexity | Agree/Disagree   - InterCARE is too complex to put into place in my clinic (H). - Including treatment partners to help participants manage both HIV and high blood pressure would be too complicated. (C, T) - Combining high blood pressure and HIV care into the same clinic visit would be too complicated. (C ) |  |
| Adaptability | Agree/Disagree   - It would be difficult to adapt InterCARE to meet the needs of different populations and groups of HIV-positive individuals with HTN in my clinic. (H) | Healthcare Providers   - “In your opinion, what kinds of changes or alterations need to be made to the components of InterCARE so it will work effectively in this clinic?” - “How well do you think InterCARE will meet the needs of the PLWH with HTN served by your Clinic? Why? Why not?” - “How well does the intervention fit with existing work processes and practices in your setting? What kinds of changes will be needed for InterCARE to work in your facility?” |
| Compatibility | Agree/Disagree   - InterCARE is compatible and consistent with the needs of HIV-positive individuals with HTN in my clinic. (H) - A treatment partner for high blood pressure will help participants remember to go to appointments. (C,T) - A treatment partner for high blood pressure will help participants remember to take their medications. (C,T) - Treatment partners will be successful in helping participants make changes to their diet to reduce blood pressure. (C,T) - Treatment partners will be successful in helping participants increase their physical activity to reduce blood pressure. (C,T) - A treatment partner will be able to teach participants about high blood pressure and how to manage their condition. (T) | Patient   - “What is your opinion about having a peer to help you with high blood pressure management? What might the benefits be? The barriers? What do you think the job of this peer should be? What aspects of high blood pressure care could they help with?”   Healthcare Provider   - “What are possible challenges compared to existing programs? Where do you think this might not work?” - “Can you describe how the intervention will be integrated into current processes?” - “Is this intervention similar to other interventions and initiatives you have been involved with before? Is this intervention similar to other projects or interventions that have been started at the clinic before? Is this intervention acceptable and in line with the values of the clinic? Is it compatible with other activities and projects currently occurring at the clinic?” |
| Relative Advantage | Agree/Disagree   - InterCARE would be more effective than interventions we are currently using to manage HTN in PLWH in my clinic. (H) | Patient:   - “Is there another program or idea you have that you think would be better than the program we are describing?”   Treatment Partner   - “In what ways do you think this approach of integrating both high blood pressure and HIV care into the hospital and your role as a treatment partner will be better than the care that is currently given? What other programs to help people with high blood pressure manage their condition have you seen? Are there any ways in which this approach could be better? In which ways will it not be as helpful?”   Healthcare Provider   - *“*How does the intervention compare to how people living with HIV and high blood pressure are managed in your clinic? What advantages does the intervention have compared to existing programs? How do you think this might help improve the care for PLWH and HTN in your clinic?” - “Will the intervention replace or complement current programs or processes?” |
| Design | Agree/Disagree   - InterCARE would be successful in improving treatment of HIV-positive individuals with HTN in my clinic. (H, T) - This program will be successful in improving participant knowledge about managing their own high blood pressure. (C) - InterCARE would be easy to understand and use after receiving training. (H) - InterCARE would have a visible and substantial impact on the health status of HIV-positive individuals with HTN in my clinic. (H) - HIV-positive individuals with HTN in my clinic would really benefit from InterCARE. (H) - Importance of provider training, electronic health record, and treatment partner. (H) | Patient, Treatment Partner, Community Members:   - “What is your opinion about the program we are proposing overall? What would make things better? What do you the challenges might be?” - “What is your opinion specifically about combining high blood pressure and HIV care into a single clinic visit? What are some of the barriers to combining high blood pressure and HIV care into a single clinic visit?”   Treatment Partner   - “In this program, we are planning to use treatment partners such as yourself for patients with both high blood pressure and HIV. These treatment partners will help patients manage both of these conditions at the same time. What are your opinions regarding the use of treatment partners in helping patients with both HIV and high blood pressure? In which ways can treatment partners be helpful for these patients? What should the role of the treatment partner be for high blood pressure? What are the challenges for treatment partners in helping patients manage both HIV and high blood pressure?” |
| **Inner Setting** | | |
| Structural Characteristics | Agree/Disagree   - InterCARE would be problematic because we do not have enough HIV medical and supportive care resources to care for any additional HIV-positive participants with HTN. (H) - InterCARE requires too many staff or other resources. (H) |  |
| Available Resources |  | Healthcare Providers   - Do you expect to have sufficient resources to implement the intervention? What resources are you counting on? Are there any other resources that you received, or would have liked to receive? What resources will be easy to procure? What resources will not be available? |
| Access to knowledge and information |  | Treatment Partner   - “What have your experiences been with getting formal training [In the hospital, training by healthcare workers] to be a treatment partner for HIV? Which sections may be helpful in training? What improvements may be made regarding training treatment partners to help manage them HIV? What kind of support (formal or informal from the community) which is ongoing is acceptable? What else can also be helpful?” - Where do you get knowledge/information about high blood pressure and ways of preventing the disease? Ways of managing high blood pressure?   Community members   - - - - 1. Where do you get your knowledge about high blood pressure and ways to prevent disease? Ways to manage high blood pressure? |
| **Individuals** | | |
| Opinion Leaders |  | Patient, Community Members   - “How should information about this intervention be communicated with you as a patient? (e.g e-mails, brochures, public announcements at churches, schools, kgotla?) Who should communicate this information to you (e.g. local leaders/chiefs, doctors, nurse)?”   Healthcare Provider   - “Who are the key influential individuals to get on board with this implementation?” - “Who are the people who usually lead new initiative to improve care? Will they be able to play that role for InterCARE? What position do these champions have in your clinic? How do you think they will help with implementation? Getting people to use the intervention?” |
| Innovation Deliverers |  | Treatment Partner   - “What qualifications or characteristics should treatment partners for hypertension have?”   Healthcare Providers   - “What kind of support or actions will be needed from leaders in your organization to help make implementation successful? Who are these leaders? How do attitudes of different leaders vary? Do they know about the intention to implement the intervention?” |
| **Outer Setting** | | |
| Local Attitudes |  | Treatment Partner, Community Members   - “We have noticed that in Botswana, patient with HIV sometimes experience stigma/discrimination. By stigma/discrimination, I mean other members of the community who discriminate against or disapprove of you if you have HIV. What has your experience been regarding HIV related stigma/ discrimination?” - “Have you encountered stigma associated with you having high blood pressure? By stigma I mean discrimination against you or disapproval of you by other people because you have high blood pressure.” |

**Table 5: Survey responses mapped to Consolidated Framework for Implementation Research (CFIR) Constructs**

|  | Healthcare Providers | Community Members | Treatment Partners |
| --- | --- | --- | --- |
| **CFIR Innovation Domain** | | | |
| **Construct: Innovation Complexity** | | | |
| InterCARE is too complex to put into place in my clinic. | 5 (25%) Agree |  |  |
| Including treatment partners to help patients manage both HIV and HTN would be too complicated. |  | 15 (38%) Agree | 8 (40%) Agree |
| Combining HTN and HIV care into the same clinic visit would be too complicated. |  | 12 (30%) Agree |  |
| **Construct: Innovation Adaptability** | | | |
| It would be difficult to adapt InterCARE to meet the needs of different populations and groups of HIV-positive individuals with HTN in my clinic. | 7 (35%) Agree |  |  |
| **Construct: Innovation Compatibility** | | | |
| InterCARE is compatible and consistent with the needs of HIV-positive individuals with HTN in my clinic. | 18 (90%) Agree |  |  |
| A treatment partner for high BP will help patients remember to go to appointments. |  | 38 (95%) Agree | 19 (95%) Agree |
| A treatment partner for high BP will help patients remember to take their medications. |  | 39 (98%) Agree | 20 (100%) Agree |
| Treatment partners will be successful in helping patients make changes to their diet to reduce BP. |  | 38 (95%) Agree | 20 (100%) Agree |
| Treatment partners will be successful in helping patients increase their physical activity to reduce BP. |  | 39 (98%) Agree | 19 (95%) Agree |
| A treatment partner will be able to teach patients about high BP and how to manage their condition. |  |  | 19 (95%) Agree |
| **Construct: Innovation Design** | | | |
| InterCARE would be successful in improving treatment of HIV-positive individuals with HTN in my clinic. | 18 (90%) Agree |  | 16 (80%) Agree |
| This program will be successful in improving patient knowledge about managing their own high BP. |  | 39 (98%) Agree |  |
| InterCARE would be easy to understand and use after receiving training. | 17 (85%) Agree |  |  |
| InterCARE would have a visible and substantial impact on the health status of HIV-positive individuals with HTN in my clinic. | 18 (90%) Agree |  |  |
| HIV-positive individuals with HTN in my clinic would really benefit from InterCARE. | 19 (95%) Agree |  |  |
| Provider Training is important. | 15 (75%) Agree |  |  |
| Electronic Health Record is important. | 13 (65%) Agree |  |  |
| Treatment Partners are important. | 16 (80%) Agree |  |  |
| **Construct: Innovation Relative Advantage** | | | |
| InterCARE would be more effective than interventions we are currently using to manage HTN in PLWH in my clinic. | 17 (85%) Agree |  |  |
| **CFIR Domain Inner Setting** | | | |
| **Construct: Structural Characteristics** | | | |
| *Work Infrastructure and Available Resources*: InterCARE would be problematic because we do not have enough HIV medical and supportive care resources to care for any additional HIV-positive patients with HTN. | 8 (40%) Agree |  |  |
| *Work Infrastructure:* InterCARE requires too many staff or other resources. | 13 (65%) Agree |  |  |

*Not all questions were answered by all groups, and certain questions were tailored for certain participant groups

**Table 6: Confidence and Training Managing HIV and HTN**

| Treatment Partner HIV Training (n=20) | | Health Provider HTN Training (n=11)* | |
| --- | --- | --- | --- |
| I have received adequate training as a HIV treatment partner | 11 (55%) Agree | Diagnosing HTN | 8 (72.7%) feel confident |
| I am confident I have the knowledge and skills required to be at treatment partner for HIV | 14 (70%) Agree | Prescribing HTN Medications | 4 (36.4%) feel confident |
| I need additional support to complete the job I am expected to do as a treatment partner for HIV | 18 (90%) Agree | Counseling participants on diet for HTN | 11 (100%) feel confident |
| I am expected to do too many things as a treatment partner for HIV | 12 (60%) Agree | Identifying when HTN is not well controlled | 11 (100%) feel confident |
|  |  | Adjusting medications when HTN is not controlled | 3 (27.3%) feel confident |

*Analysis limited to healthcare providers directly responsible for managing HTN (nurses, family nurse practitioners, and doctors)

**Table 7: Additional Qualitative Themes and Quotations**

| **Theme** | **Interviewee Demographics** | **Quote** |
| --- | --- | --- |
| **Outer Setting Domain** | | |
| ***Local Attitudes: HIV Stigma, HTN Stigma***  Some note HIV stigma to be a major challenge to care-seeking behaviors, especially since certain clinic structures do not allow patients with HIV to remain anonymous.  HIV stigma may prevent successful treatment partner selection as some patients are unwilling to disclose their HIV status to their peers and some treatment partners are afraid of their HIV status being disclosed.  Participants note that there is no stigma towards high blood pressure and perhaps HIV stigma is no longer the major issue it used to be. | Female community member  Female treatment partner  Male with HIV and HTN | *"I have noticed that at hospitals they are being separated from the others, like when you get to the hospital it looks obvious as to which consultation they are queuing for."*  *“Yes, stigma is very common... Even at my household there is so much stigma, I always hear [my family] criticising people living with HIV [which] is why I opted to open up only to my child because I wanted to avoid their stressful comment[s]”*  *“No sir there is no stigma [against high blood pressure]…this thing is now common [and is the] same as taking ART or taking any pills…You see when AIDS started [there was stigma]. These days a person can go in public saying “I’m going to charge meaning ART’* |
| **Inner Setting Domain** | | |
| ***Structural Characteristics (Work Infrastructure):***  There were mixed sentiments regarding the burden of clinical care integrating HIV and HTN services would provide.  Providers felt that InterCARE was feasible as there were already several structures in place to facilitate integration, including a leadership structure within the clinic to organize and implement the intervention components.  Nurses typically led new disease management initiatives as doctors or FNPs are not always readily available. | Male nurse  Male public health  officer  Male nurse  Male nurse | *" [Measuring and entering clinical data for patients enrolled in InterCARE may take] double or triple [the] time of any other general patients so I believe some other programs [patients without HIV and HTN] might end up believing that they are getting delayed unnecessarily."*  *“We [as healthcare providers] deal with patients having to come for a certain service, and tomorrow they are coming for a different service…but knowing that [a patient] might come here once and still be able to get help for 2 or 3 ailments [could work for our staff and prevent overworking and allow them to utilize their clinic time more efficiently.”*  *"We have leaders like district leaders, matrons, chief doctors, also [there] are cluster matrons and facility matrons…They will be able to take the rightful administrative steps…in order for the program to run smoothly"*  *"we don’t have a medical doctor in the cluster so I would say nurses are the ones who have the lead for the new initiatives of improving the care"* |
| ***Available Resources:***  Providers commented on the challenges of seeing participants in a timely fashion due to the caseload.  Lack of equipment and power outages may make it difficult to implement InterCARE, especially since the automatic blood pressure cuffs used in clinic relied on electricity. | Male public health  officer  Male nurse | *"[As] health care providers we deal with [patients having to come for different services]. Tomorrow, they are coming for a different service, and then a third time. Knowing that I [as a patient] might come here once and still be able to get help for 2 or 3 ailments [would benefit the staff]. It [is difficult to be] overworked.”*  *"We having been reporting that we having defunct bathroom scale…[and] we work with only one [BP] monitor, the other one is not working.” "the[re] is a regular cut of electricity [and] since our BP machines rely on electricity it means that the checking of BP and pulse rate may be compromised"* |
| Medication stock outs and distance to the pharmacy to pick up medications is a barrier to medication adherence.  There is a lack of access to healthy food options due to cost and availability. | Male with HIV and HTN  Female treatment partner | *“…sometimes you will go to a clinic where the medication is out of stock and you end up going to X where the medication will also be out of stock and you end up buying or ringing the clinic number to ask if the medication is available. Other challenges could be… travelling a long distance to come for [a] medication refill.”*  *“These foods we are eating, like morogo (spinach) is sometimes very difficult to [have available] and some foods [we are told to eat] like apples, I don’t manage to buy.”* |
| ***Access to knowledge and information:***  Providers requested more dedicated HTN management training.  Treatment partners were more knowledgeable on HIV than HTN. They understood the importance of medication adherence and diet but couldn’t always describe the causes and risk factors | Male nurse  Female treatment partner | *“For me I believe [there can be a] large scale training for dispenser[s] and nurses in the clinic. Maybe you can lobby for it or recommend [it].”*  *"High blood pressure is plenty…I do not know whether [it] is [a] sickness or [if] it’s associated with family, I really do not know.”* |
| ***Compatibility:***  The definition of a treatment partner is broad in practice and accepted as a role many individuals take on voluntarily for a wide variety of medical conditions.  Both patients with HIV and treatment partners look favorably on the treatment partner model and have had positive experiences with the support they have received. | Male nurse  Male with HIV and HTN  Female treatment partner | *"for instance if somebody has been involved in a road traffic accident and has a fracture… [relatives or close partners take care of that patient so a treatment partner is used for many conditions].*  *"I do not have any challenges since my treatment partner gives me full support towards my diet, what to/what not to do."*  *"I have learnt certain things from my partner. Since I became his treatment partner he has [taken his pills on time]. He is the kind of person who knows what is supposed to do and not do…"* |
| **Individuals Domain** | | |
| ***Opinion Leaders:***  Liaising with the village chiefs and village health care committee will improve acceptance and adoption. | Male public health officer | *"The Kgosi are the gate keepers to the village so if they are receptive of initiative, chances are people are going to be [accepting]…”* |
| ***Innovation Recipients:***  It is important to sensitize participants so that patients will be accepting of InterCARE. Participants recommended the information being delivered by healthcare providers. | Male public health officer  Female with HIV and HTN | *“People are resistant to change so if they are not aware of the new initiative. People will always be receptive if they have been taught and sensitized and given [reasons for why they must] change their traditional way of doing things.”*  *“I prefer the healthcare workers for I know them.”* |
| ***Implementation Facilitators:***  For both HIV and HTN care, champions, patients engaged in the healthcare system and model medication adherence healthy lifestyle behaviors, are natural advocates. | Male nurse | *"They [model patients] are in a position to share their personal experiences [and positively influence their peers]. [Their peers] might be paranoid [about this new thing, but will join if they see that other members in the community are participating].* |

KEY INFORMANT INTERVIEW GUIDES

**Patient Key Informant Interview**

***SECTION ONE***

Do I have your permission to interview you regarding your thoughts on high blood pressure management and the intervention we are planning?

Do I have your permission to record this interview? *(If YES, turn on digital recorder and state participant ID before starting the interview)*

***PARTICIPANT BACKGROUND INFORMATION***

**First, we would like to know a little about you.**

1. Age……………….
2. Gender: a. Female b) Male c) Other d) Refuse to answer
3. Highest level of school completed
   1. Less than Primary (Including Non formal)
   2. Primary School (Standard 1-7)
   3. Junior Secondary (Form 1-3)
   4. Senior Secondary (Form 4-5)
   5. Higher than senior secondary (university, diploma, etc.)
4. . What is the main source of energy used for cooking?
5. Charcoal/wood
6. Paraffin
7. Gas
8. Electricity (mains)
9. Electricity (solar)
10. No cooking done
11. Other
12. Does any member of this household have any of the following that are currently working? (check all that apply).

- Radio
- Don't want to answer
- TV
- Landline telephone
- Cell phone
- Computer
- Access to internet
- Refrigerator

6. Does any member of this household (excluding visitors) own any of the following forms of transport in working condition? (check all that apply).

- Motor vehicle (car, truck, taxi, etc)
- Tractor
- Bicycle
- Motorcycle/scooter
- Donkey or cow cart
- Donkey/horses

***PATIENT BACKGROUND INFORMATION***

**We will now ask you some questions about the services you have received at this clinic in the past.**

1. Where do you go most frequently to get HIV care?
2. What have your experiences been working with a treatment partner (Mopati) for your HIV care?
   1. What are the benefits?
   2. What are the challenges?
3. What is your relationship to your treatment partner (e.g Is he or she a family member or close friend/acquaintance/household member)?
   1. What are some of the benefits of having this type of relationship/connection with your treatment partner?
   2. What are some of the challenges?
4. What have your experiences been getting counseling at the clinic for your HIV?
   1. What have been the benefits of HIV counseling?
   2. What improvements can be made regarding HIV counseling?
5. Where do you go most frequently to get care for your high blood pressure? Is it the same location as where you get HIV care? ***SECTION TWO:***

***We will now ask you some questions regarding your diagnosis of HTN and experiences having HTN.***

1. When were you diagnosed with high blood pressure and how was the diagnosis made?
   1. *Probe: [What was your experience? How was the diagnosis communicated to you? How easily did you understand this diagnosis?]*
2. What are some things that have made it easier and helped you to manage your high blood pressure?
3. What are some of the challenges you face in managing your high blood pressure?
4. How has it been taking blood pressure medications? If not taking medications, can skip?
   1. What has made it easy or hard to get your medications
5. Who gives you information and support in the clinic to manage your blood pressure?
   1. Probe: Do you spend enough time with this person (nurse or doctor)?
   2. How would you describe your interactions? Have they been beneficial? Have they helped you follow the recommendations for managing high blood pressure?
6. Who is a person at home or in your community that you rely on to help you manage your blood pressure [can be more than one person]? Probe: Is he or she your Mopati for HIV?
   1. What aspects of your high blood pressure care does this person help you with? What are the benefits of having a person to help you manage your blood pressure?
   2. Are there aspects of your high blood pressure care this person is unable to help you with?
7. What have your experiences been getting counseling for your high blood pressure at this clinic in the past 12 months? *[Prompt: This counseling may be related to lifestyle changes to manage your high blood pressure (diet modifications, increasing physical activity) or if you are on medications - taking your medications regularly and monitoring for side effects]*
   1. What have been benefits of this counseling? What improvements can be made regarding high blood pressure management counseling?
8. How do you get care for both HIV and high blood pressure?
   1. *Probe: Is this done at the same time in a single clinic visit? In a single day? By the same provider?*
   2. How is the care you get for HIV and high blood pressure similar or different?
      1. *[Probe: Is getting medications for HIV and high blood pressure similar or different?]*
      2. *[Probe: Is the way you get counseling similar or different?]*
9. *[For this question, you want to probe the patient based on their responses to the question above. Example: If they responded that they receive HIV and HTN care at different clinic locations, different clinic visits, or by different providers, you can ask if they would prefer receiving care for both diseases in the same visit].* We would like to know more about how you receive care for both of your HIV and high blood pressure.
   1. What works well about how you are receiving HIV care? High blood pressure care? How well coordinated are they (by coordination we mean receiving HIV and high blood pressure care in the same clinic visit by the same provider; also, receiving medications for both medical conditions at the same pharmacy)
   2. *[Probe: Do you prefer that they are separated with different providers? Or, would you prefer to see one provider who manages and counsels you on both HIV and high blood pressure in the same clinic visit?]*

Next, we are interested in finding out your views of the InterCare intervention presented. Specifically, whether you think it might be an improvement in how you are currently getting care and support to help you manage your HIV and high blood pressure; To remind you, InterCARE is a program that will integrate care for high blood pressure with HIV care. You will see a single provider in the same clinic visit to receive care for both HIV and high blood pressure. The major components of this intervention include: 1) Training and education for health care providers on hypertension and cardiovascular disease. 2) An electronic medical record that will help health care providers keep track of the blood tests you require and when you need refills on your medications. 3) Using a Mopati to help you manage high blood pressure and HIV.

1. What is your opinion about the program we are proposing overall? What would make things better? What do you the challenges might be?
   1. *[Probe: What might the benefits be to you? The barriers? What should the goals of this program be?]*
   2. *[Probe: Do you think this program will improve access to services? Improve access to doctors and nurses? Reduce wait times? Help reduce travel time and expense? Improve patient education and counseling? ]*
2. What is your opinion specifically about combining high blood pressure and HIV care into a single clinic visit?
   1. *[Probe-How would this be a change from how you are currently receiving care? Would it be better if it is? If not a change, what are other things that would be helpful in improving your high blood pressure and HIV care?]*
   2. What are some of the barriers to combining high blood pressure and HIV care into a single clinic visit?
3. We will now ask a couple questions about your opinion on peers to support high blood pressure management.
   1. What is your opinion about having a peer to help you with high blood pressure management? What might the benefits be? The barriers? What do you think the job of this peer should be? What aspects of high blood pressure care could they help with?
4. We have described the program we are planning on starting in this clinic. What else could make it easier for you to receive care for and manage your HIV and your blood pressure?

***SECTION FOUR***

***Through these* final questions, we would like to find out how this intervention will affect work processes in your facility. We are also interested in your opinion of whose buy-in, input and expertise is needed to implement and maintain the intervention.**

1. How should information about this intervention be communicated with you as a patient? (e.g e-mails, brochures, public announcements at churches, schools, kgotla?)
   1. Who should communicate this information to you (e.g. local leaders/chiefs, doctors, nurse)?
2. Is there another program or idea you have that you think would be better than the program we are describing?
   1. [If Yes] Can you describe this program or idea?
   2. [If Yes] Why would people prefer the alternative program or idea?
3. We are about to finish the interview. Is there anything else you would like to add about the proposed program to improve both HIV and high blood pressure care in this clinic?

**End of the Interview. Thank you for participating in this interview.**

**Treatment Partner Key Informant Interview**

***SECTION ONE***

Do I have permission to record this interview/discussion? *(If its yes, switch on the voice recorder and state the participants number before starting the interview)*

***INFORMATION ABOUT PARTCIPANT***

**Firstly, we would like to know a little bit about you**

1. Age ……………….
2. Gender: a. Female b) Male c) Other d) Prefer not to answer
3. Highest level of education completed
   1. Less than Primary (Including non-formal education)
   2. Primary School (Standard 1-7)
   3. Junior Secondary school (Form 1-3)
   4. Senior Secondary school (Form 4-5)
   5. Higher than senior secondary school (university, diploma, etc.)
4. Are you currently employed?
   1. Yes [skip to question 5]
   2. No [skip to question 6]
5. In your main job what kind of work do you do?
   1. Temporary/casual employment
   2. Formal employment (full-time)
   3. Formal employment (part-time)
   4. Self-employed in agriculture
   5. Self-employed, generation income, full time
   6. Self -employed, generating income, part time
   7. Other
6. What are the reasons why you are unemployed?
   1. I am waiting to continue agricultural work
   2. I am unemployed (job hunting)
   3. I am unemployed (waiting to start a new job)
   4. I am unable to work (permanently ill or injured)
   5. Student/trainee/volunteer
   6. Housewife/home caretaker (not looking for a job)
   7. Retired
   8. Other
   9. Not looking for a job
7. How long have you been a treatment partner for HIV?
   1. First time
   2. < 1 year
   3. 1-2 years
   4. 3-5 years
   5. > 5 years

***SECTION TWO***

***These next questions will help us understand* why it is important to do this program now; and whether it address a current gap in the patient care system in your health facility**

***Firstly, we will ask you a few questions about your experience as a treatment partner for HIV***

1. How has your experience been working as a treatment partner for an individual who is living with HIV
   1. What kind of activities do you do in this role?
   2. What are the benefits? What are the challenges?
2. What is your relationship with the person living with HIV who you are working with (example is he/she a family member or a close friend/acquaintance)?
   1. What are some of the benefits of this kind of relationship/connation with an individual with HIV who you are working with?
   2. What are some of the challenges?
3. How has the hospital supported your work as a treatment partner?
   1. Was it useful?
   2. What else can be offered by the hospital to support you in this role?
4. We have noticed that in Botswana, patient with HIV sometimes experience stigma/discrimination. By stigma/discrimination, I mean other members of the community who discriminate against or disapprove of you if you have HIV. What has your experience been regarding HIV related stigma/ discrimination?
   1. What are the challenges associated with HIV related stigma? For treatment partners?
   2. Do you have any suggestions about what can be done by the hospital to reduce this stigma?
5. What have your experiences been with getting formal training [In the hospital, training by healthcare workers] to be a treatment partner for HIV?
   1. Which sections may be helpful in training?
   2. What improvements may be made regarding training treatment partners to help manage them HIV
   3. What kind of support (formal or informal from the community) which is ongoing is acceptable? What else can also be helpful?
      1. Do you receive any feedback from the hospital on how you are doing as treatment partner?
      2. Do you receive any feedback from the person you are supporting?

***As explained, we are working to expand the Mopati program to support people living with HIV who also have high blood pressure. I will now move on to asking you about your experiences and knowledge about high blood pressure in the community.***

1. How common is high blood pressure in your community? How do you know this?
   1. Do you know people with hypertension? How difficult is it or them to control their blood pressure?
   2. Where do you get knowledge/information about high blood pressure and ways of preventing the disease? Ways of managing high blood pressure?
2. What are your personal experiences with high blood pressure?
   1. Exclude yourself? Your family member? Friends?
   2. If it is yes to having personal experience, what has been helpful and made it easy for them [or you] to manage blood pressure?
   3. If yes to having personal experience, what are the challenges to managing blood pressure?
3. Have you encountered stigma associated with you having high blood pressure? By stigma I mean discrimination against you or disapproval of you by other people because you have high blood pressure.
   1. If yes, what can be done to decrease this stigma in the community? At the clinic?

SECTION THREE

Next, we are interested in finding out your views of the InterCare program mentioned. Specifically, whether it is better compared to what you are currently doing to manage PLHIV and HTN; and if it will need to be adapted so that it works in this facility. To remind you, InterCARE is a program that will integrate care for high blood pressure with HIV care. You will see a single provider in the same clinic visit to receive care for both HIV and high blood pressure. The major components of this intervention include: 1) Training and education for health care providers on hypertension and cardiovascular disease. 2) An electronic medical record that will help health care providers keep track of the blood tests which your peer requires and when your peer needs refills on your medications. 3) Using a Mopati, such as yourself to help manage high blood pressure and HIV.

1. In this program, we are planning to use treatment partners such as yourself for patients with both high blood pressure and HIV. These treatment partners will help patients manage both of these conditions at the same time. What are your opinions regarding the use of treatment partners in helping patients with both HIV and high blood pressure?
   1. In which ways can treatment partners be helpful for these patients?
   2. What should the role of the treatment partner be for high blood pressure?
      1. *Probe: Management of hypertension has many different components which includes ensuring that patients eat a healthy diet, increase their physical activity and stop smoking or drinking alcohol. Should a treatment partners be expected to help patients make these lifestyle changes when managing high blood pressure?*
   3. What are the challenges for treatment partners in helping patients manage both HIV and high blood pressure?
   4. In addition to the training which will be provided, what kind of support would you need to add hypertension support to the support you are already giving for HIV?
      1. *Probe- What type of knowledge would you need?*
      2. *What type of training?*
      3. *What type of supervision if any supervision?*
      4. *Other needs or types of support?*
2. What qualifications or characteristics should treatment partners for hypertension have?
   1. *Probe – Prior experience with managing hypertension? Could be related to or live in the same household as the patient?*
3. What kind of feedback would be helpful to receive as a Mopati when helping someone manage their HIV and high blood pressure?
   1. *Probe-how they are doing? How well controlled is their blood pressure? Have they gained any knowledge in managing their blood pressure?*
4. What are your overall thoughts about the proposed program?
   1. After hearing about the three components/sections, are there any sections which you would like to change?
      1. Add on? Get rid of?
   2. What are you worried about regarding this program?
5. In what ways do you think this approach of integrating both high blood pressure and HIV care into the hospital and your role as a treatment partner will be better than the care that is currently given?
   1. What other programs to help people with high blood pressure manage their condition have you seen?
   2. Are there any ways in which this approach could be better? In which ways will it not be as helpful?

***SECTION FOUR***

***Through these* final questions, we would like to find out how this program will affect work processes in your facility. We are also interested in your opinion of whose buy-in, input and expertise is needed to implement and maintain the program.**

1. How should information about this program be communicated to you as a treatment partner? (example: e-mails, brochures/pamphlets, public announcements at churches, schools, kgotla?)
   1. Who will communicate this information to members of the community (example: local leaders/chiefs, doctors, nurse)?
2. We are about to complete the interview. Is there anything else you would like to add about the proposed program to improve both HIV and high blood pressure care in this hospital?

**Healthcare Provider Key Informant Interview**

***SECTION ONE***

Do I have your permission to record this interview? *(If YES, turn on digital recorder and state participant ID before starting the interview)*

***PARTICIPANT BACKGROUND INFORMATION***

**First, we would like to know a little about you.**

1. Gender: a) Female b) Male c) Other d) Refuse to answer
2. Age……………………………
3. Role at Health Facility

a) Medical Officer

b) FNP

c)Nurse

d)Dietician

e) Pharmacist

f) Other (please specify) …………….

1. Highest Academic Qualification: ……………………………………………
2. Years working at the Clinic………………………………………….
3. List on-job Hypertension training you received in past 2years.

***SECTION TWO***

Next, we are interested in finding out your views of the InterCare intervention presented. Specifically, whether it is better compared to what you are currently doing to manage PLHIV and HTN; and if it will need to be adapted so that it works in this facility.

1. How does the intervention compare to how people living with HIV and high blood pressure are managed in your clinic?
   1. What advantages does the intervention have compared to existing programs?
      1. How do you think this might help improve the care for PLWH and HTN in your clinic?
   2. What are possible challenges compared to existing programs?
   3. Where do you think this might not work?
2. How much of a problem is hypertension diagnosis, management, and care in this clinic?
   1. How does this compare to other areas where change is needed?
   2. How important is it to others, such as your coworkers or leaders, to implement the intervention compared to the other priorities?
3. What outcomes (e.g. patient blood pressure, documentation in the EHR, knowledge assessments for providers) would be helpful to measure to convince staff and patients that this intervention is useful and should be continued?
   1. What outcomes would be important for co-workers?
   2. What outcomes would be important for administrative leaders?
   3. What outcomes would be important for patients?
4. In your opinion, what kinds of changes or alterations need to be made to the components of InterCARE so it will work effectively in this clinic?
   1. Specifically, what changes in training?
   2. EMR Implementation?
   3. Use of treatment partner?

***SECTION THREE***

***These next questions will help us understand* why is it important to do this intervention now; and whether it address a current gap in the patient care system in your health facility**

1. How well do you think InterCARE will meet the needs of the PLWH with HTN served by your Clinic?
   1. Why?
   2. Why not?
   3. In what ways will the intervention meet their needs? E.g. improved access to services? Reduced wait times? Help with self-management? Reduced travel time and expense?
2. How do you think the individuals served by your clinic will respond to the use of the treatment partners?
   1. What will help them participate?
   2. What are some of the barriers?

***SECTION FOUR***

**Through these next set of questions, we are interested in finding out if the intervention will fit well in your system and if it’s feasible to implement it now.**

1. How well does the intervention fit with existing work processes and practices in your setting?
   1. What kinds of changes will be needed for InterCARE to work in your facility?
   2. Specifically, would such changes involve:
      1. Changes in scope of practice of individuals? Task sharing?
      2. Changes in formal policies?
      3. Patient flow
      4. Other?
   3. What are likely issues or complications that may arise?
2. Can you describe how the intervention will be integrated into current processes?
   1. How will it interact or conflict with current programs or processes?
3. Will the intervention replace or complement current programs or processes?
   1. In what ways?
4. Do you expect to have sufficient resources to implement the intervention?
   1. [If Yes] What resources are you counting on? Are there any other resources that you received, or would have liked to receive?
   2. What resources will be easy to procure?
   3. [If none] What resources will not be available?
5. Is this intervention similar to other interventions and initiatives you have been involved with before?
   1. Is this intervention similar to other projects or interventions that have been started at the clinic before?
   2. Is this intervention acceptable and in line with the values of the clinic?
   3. Is it compatible with other activities and projects currently occurring at the clinic?
6. Is there a difference between programs started by outside organizations (Botswana Harvard Partnership, University of Wisconsin) and programs started by staff at a clinic?
7. What kind of support or actions will be needed from leaders in your organization to help make implementation successful?
   1. Who are these leaders? How do attitudes of different leaders vary?
   2. Do they know about the intention to implement the intervention?
   3. What kind of support do you need going forward?
   4. What types of barriers might they create?

***SECTION FIVE***

***Through these* final questions, we would like to find out how this intervention will affect work processes in your facility. We are also interested in your opinion of whose buy-in, input and expertise is needed to implement and maintain the intervention.**

1. Who are the key influential individuals to get on board with this implementation?
2. Who are the people who usually lead new initiative to improve care?
   1. Will they be able to play that role for InterCARE?
   2. What position do these champions have in your clinic?
      1. How do you think they will help with implementation? Getting people to use the intervention?
3. We are about to end the interview. Are there any other additional comments you would like to add?

**End of the Interview. Thank you for participating in this interview.**

**Community Member Key Informant Interview**

***SECTION ONE***

Do I have your permission to interview you regarding your thoughts on high blood pressure management and the intervention we are planning? Do I have your permission to record this interview? *(If YES, turn on digital recorder and state participant ID before starting the interview)*

***PARTICIPANT BACKGROUND INFORMATION***

**First, we would like to know a little about you. Can skip if quantitative survey was completed.**

- - - 1. Age……………….
      2. Gender: a. Female b) Male c) Other d) Refuse to answer
      3. What is your role in the community?
         1. Community leader
         2. Consumer Services
         3. Local council
      4. Highest level of school completed
         1. Less than Primary (Including Non formal)
         2. Primary School (Standard 1-7)
         3. Junior Secondary (Form 1-3)
         4. Senior Secondary (Form 4-5)
         5. Higher than senior secondary (university, diploma, etc.)
      5. What is the main source of energy used for cooking?
         1. Charcoal/wood
         2. Paraffin
         3. Gas
         4. Electricity (mains)
         5. Electricity (solar)
         6. No cooking done
         7. Other
      6. Does any member of this household have any of the following that are currently working? (check all that apply).
         1. Radio
         2. TV
         3. Landline telephone
         4. Cell phone
         5. Computer
         6. Access to internet
         7. Refrigerator
         8. Don't want to answer
      7. Does any member of this household (excluding visitors) own any of the following forms of transport in working condition? (check all that apply).
         1. Motor vehicle (car, truck, taxi, etc)
         2. Tractor
         3. Bicycle
         4. Motorcycle/scooter
         5. Donkey or cow cart
         6. Donkey/horses

***SECTION TWO***

***These next questions will help us understand* why is it important to do this program now; and whether it address a current gap in the patient care system in your health facility**

***AS described, we are working to expand the Mopati program to support people living with HIV who also have high blood pressure. I will now move on to asking you about your experiences and knowledge about high blood pressure in the community.***

- - - 1. How common is high blood pressure in your community? How do you know this?
         1. Do you know people with high BP? How hard is it for them to control their BP?
         2. Where do you get your knowledge about high blood pressure and ways to prevent disease? Ways to manage high blood pressure?
      2. What is your personal experience with high blood pressure?
         1. Ex. Yourself? Someone in your family? Friends?
         2. If yes to having personal experience, what has been helpful and made it easier for them [or you] to manage blood pressure?
         3. If yes to having personal experience, what are challenges to managing blood pressure?

***SECTION THREE***

Next, we are interested in finding out your views of the InterCare intervention presented. Specifically, whether you think it might be an improvement in how you are currently getting care and support to help you manage your HIV and high blood pressure; To remind you, InterCARE is a program that will integrate care for high blood pressure with HIV care. You will see a single provider in the same clinic visit to receive care for both HIV and high blood pressure. The major components of this intervention include: 1) Training and education for health care providers on hypertension and cardiovascular disease. 2) An electronic medical record that will help health care providers keep track of the blood tests you require and when you need refills on your medications. 3) Using a Mopati to help you manage high blood pressure and HIV.

- - - 1. What is your opinion about the program we are proposing overall? What would make things better? What do you think the challenges might be?
         1. *[Probe: What might the benefits be to you? The barriers? What should the goals of this program be?]*
         2. *[Probe: Do you think this program will improve access to services? Improve access to doctors and nurses? Reduce wait times? Help reduce travel time and expense? Improve patient education and counseling?]*
      2. What is your opinion specifically about combining high blood pressure and HIV care into a single clinic visit?
         1. *[Probe-How would this be a change from how the clinic is currently providing care? Would it be better if it is? If not a change, what are other things that would be helpful in improving your high blood pressure and HIV care?]*
  1. What are some of the barriers to combining high blood pressure and HIV care into a single clinic visit?

1. We will now ask a couple questions about your opinion on peers to support high blood pressure management.
   1. What is your opinion about having a peer to support patients with high blood pressure. What might the benefits be? The barriers? What do you think the job of this peer should be? What aspects of high blood pressure care could they help with?
2. We have described the program we are planning on starting in this clinic. What else could make it easier for the community to receive care for and manage HIV and your blood pressure?
3. Do you think community members with HIV encounter stigma associated with them having HIV? By stigma, I mean other community members discriminating against or disapproving of you if you have HIV. What have your experiences been with HIV related stigma?
   1. What are the challenges associated with HIV related stigma for patients? For treatment partners?
   2. Do you have any suggestions on things that can be done by the clinic to minimize this stigma?
4. Do you think community members with high blood pressure encounter stigma associated with them having high blood pressure? By stigma I mean discrimination against them or disapproval of them by other people because they have high blood pressure.
   1. If yes, what can be done to decrease this stigma in the community?

***SECTION FOUR***

***Through these* final questions, we would like to find out how this intervention will affect work processes in your facility. We are also interested in your opinion of whose buy-in, input and expertise is needed to implement and maintain the intervention.**

1. How should information about this intervention be communicated with community members? (e.g e-mails, brochures, public announcements at churches, schools, kgotla?)
   1. Who should communicate this information to community members (e.g. local leaders/chiefs, doctors, nurse)?
2. Is there another program or idea you have that you think would be better than the program we are describing?
   1. [If Yes] Can you describe this program or idea?
   2. [If Yes] Why would people prefer the alternative program or idea?
3. We are about to finish the interview. Is there anything else you would like to add about the proposed program to improve both HIV and high blood pressure care in this clinic?

**End of the Interview. Thank you for participating in this interview.**

**Survey Tools**

**Patient Survey**

Goal of the survey:

- Understand the experience of patients related to HIV and HTN
- Understand what they view as areas for improvement in HTN management (may or may not be related to InterCARE)
- Explore their attitudes towards integrating Mopati for HIV and HTN

**SECTION 1: Baseline Information**

- - - 1. Age……………….
      2. Gender: a. Female b) Male c) Other d) Refuse to answer
      3. Highest level of school completed
         1. Less than Primary (Including Non formal)
         2. Primary School (Standard 1-7)
         3. Junior Secondary (Form 1-3)
         4. Senior Secondary (Form 4-5)
         5. Higher than senior secondary (university, diploma, etc.)

1. What is the main source of energy used for cooking?

Charcoal/wood

Paraffin

Gas

Electricity (mains)

Electricity (solar)

No cooking done

Other

1. What is the main toilet facility used in this household?
2. Pit latrine within plot
3. Flush toilet within plot
4. Neighbor’s flush toilet
5. Neighbors pit latrine
6. Communal flush toilet
7. Communal pit latrine
8. Pail bucket latrine
9. Bush
10. River or other body of water
11. Other
12. Does any member of this household have any of the following that are currently working? (check all that apply).
    1. Radio
    2. TV
    3. Landline telephone
    4. Cell phone
    5. Computer
    6. Access to internet
    7. Refrigerator
    8. Don't want to answer
13. Does any member of this household (excluding visitors) own any of the following forms of transport in working condition? (check all that apply).
    1. Motor vehicle (car, truck, taxi, etc)
    2. Tractor
    3. Bicycle
    4. Motorcycle/scooter
    5. Donkey or cow cart
    6. Donkey/horses
14. Are you currently working?
    1. Yes – Question 4
    2. No – Question 5
15. In your main job what type of work do you do?
    1. Occasional or Casual employment (piece job)
    2. Seasonal employment
    3. Formal wage employment (full-time)
    4. Formal wage employment (part-time)
    5. Self-employed in agriculture
    6. Self-employed making money, full time
    7. Self-employed making money, part time
    8. Other
16. What is the reason why you are not working?
    1. Waiting to continue agricultural work
    2. Unemployed (looking for work)
    3. Unemployed (waiting to start new work)
    4. Unable to work (permanently sick or injured)
    5. Student/ Apprentice/ Volunteer
    6. Housewife/ Homemaker (not looking for work)
    7. Retired
    8. Other
    9. Not looking for work
17. Monthly Household Income
    1. < 1000
    2. 1001 – 5000
    3. 5001 - 10,000
    4. 10,001 & above
    5. Prefer not to answer
    6. I don’t know
18. Number of members (including participant) living in the household? __________
19. When was the last time you visited this clinic?
    1. This is my first time
    2. Less than 30 days ago
    3. 1-6 Months back
    4. 7-12 Months back
    5. More than 12 months back
    6. I don’t know
20. How many years have you been visiting this clinic? _____________________
21. Do you visit any other clinic for you HIV Care?
    1. Yes. List which ones___________________________
    2. No
    3. I don’t know
22. Do you visit any other clinic for you high blood pressure care?
    1. Yes. List which ones___________________________
    2. No
    3. I don’t know

**SECTION 2: HIV Care**

1. Do you have a treatment partner (Mopati) who helps you with your HIV management?
   1. Yes
   2. No
   3. I don’t know
2. How many years has this person been your treatment partner for HIV?
   1. < 1 year
   2. 1-2 years
   3. 3-5 years
   4. > 5 years
3. Do you live in the same household as your treatment partner?
   1. Yes
   2. No
4. What is your relationship to your treatment partner?
   1. Spouse (Husband or Wife)
   2. Child (Son or Daughter)
   3. Parent (Mother or Father)
   4. Sibling (Brother or Sister)
   5. Other Blood Relative (Aunt, Uncle, Grandparent, Cousin)
   6. Friend/Acquaintance
   7. Other __________________
5. Have you had other treatment partners before your current treatment partner?
   1. Yes, how many?_______
   2. No
   3. I don’t know

**SECTION 3: High Blood Pressure (HTN) Care**

1. Have you been prescribed medications for high blood pressure by a doctor or nurse?
   1. Yes
   2. No
   3. I don’t know
2. Have you ever been counselled about salt intake by a health care worker in the past 3 years?
   1. Yes
   2. No
   3. I don’t know
3. Have you ever used ANY tobacco products (including snuff)?
   1. Never
   2. Prior
   3. Current
4. If yes to any current or prior use of tobacco products, have you been counselled about tobacco cessation / not taking up tobacco use by a healthcare worker in the past 3 years?
   1. Yes
   2. No
   3. I don’t know
5. Have you ever been counselled about what weight you should aim for by a health care worker in the past 3 years?
   1. Yes
   2. No
   3. I don’t know
6. Have you ever been counselled about the amount of physical activity (or exercise) to maintain by a healthcare worker in the past 3 years?
   1. Yes
   2. No
   3. I don’t know
7. Have you ever been counselled about correct alcohol intake by a healthcare worker in the past 3 years?
   1. Yes
   2. No
   3. I don’t know
8. Have you ever had a blood test for high cholesterol in the past 3 years?
   1. Yes
   2. No
   3. I don’t know
9. Have you ever had blood test for sugar diabetes in the past 3 years?
   1. Yes
   2. No
   3. I don’t know

**Treatment Partner Survey**

**SECTION 1: Baseline Information**

Age………………

Gender: a. Female b) Male c) Other d) Refuse to answer

Highest level of school completed

Less than Primary (Including Non formal education)

Primary School (Standard 1-7)

Junior Secondary (Form 1-3)

Senior Secondary (Form 4-5)

Higher than senior secondary (university, diploma, etc.)

- - - 1. Are you currently working?
         1. Yes [jump to question 5]
         2. No [jump to question 6]
      2. In your main job what type of work do you do?
         1. Occasional or Casual employment (piece job)
         2. Seasonal employment
         3. Formal wage employment (full-time)
         4. Formal wage employment (part-time)
         5. Self-employed in agriculture
         6. Self-employed making money, full time
         7. Self-employed making money, part time
         8. Other
      3. What is the reason why you are not working?
         1. Waiting to continue agricultural work
         2. Unemployed (looking for work)
         3. Unemployed (waiting to start new work
         4. Unable to work (permanently sick or injured)
         5. Student/ Apprentice/ Volunteer
         6. Housewife/ Homemaker (not looking for work)
         7. Retired
         8. Other
         9. Not looking for work
      4. Monthly Household Income

1. < 1000
2. 1001 – 5000
3. 5001 - 10,000
4. 10,001 & above
5. Prefer not to answer
6. I don’t know
   - - 1. Number of members (including participant) living in the household? __________
       2. Does any member of this household have any of the following that are currently working? (check all that apply).
   1. Radio
   2. TV
   3. Landline telephone
   4. Cell phone
   5. Computer
   6. Access to internet
   7. Refrigerator
   8. Don't want to answer
      - 1. Does any member of this household (excluding visitors) own any of the following forms of transport in working condition? (check all that apply).
           1. Motor vehicle (car,truck,taxi, etc)
           2. Tractor
           3. Bicycle
           4. Motorcycle/scooter
           5. Donkey or cow cart
           6. Donkey/horses
        2. What is the main source of energy used for cooking?
7. Charcoal/wood
8. Paraffin
9. Gas
10. Electricity (mains)
11. Electricity (solar)
12. No cooking done
13. Other
    - - 1. How many years have you been a treatment partner for HIV?
           1. < 1 year
           2. 1-2 years
           3. 3-5 years
    1. > 5 years
       - 1. Do you live in the same household as your peer with HIV and high blood pressure?
            1. Yes
            2. No
         2. What is your relationship to your peer with HIV?
            1. Spouse (Husband or Wife)
            2. Child (Son or Daughter)
            3. Parent (Mother or Father)
            4. Sibling (Brother or Sister)
            5. Other Blood Relative (Aunt, Uncle, Grandparent, Cousin)
            6. Friend/Acquaintance
            7. Other __________________

**SECTION 2: HIV Care: LEAD IN QUESTIONS**

- - - 1. For the following section, read the statement and inform us if you: Strongly agree, Agree, Neither Agree or Disagree, Disagree, strongly disagree

|  | Strongly Agree | Agree | Neither Agree or Disagree | Disagree | Strongly Disagree | No Answer |
| --- | --- | --- | --- | --- | --- | --- |
| I have received adequate training as a HIV treatment partner |  |  |  |  |  |  |
| I am confident I have the knowledge and skills required to be at treatment partner for HIV |  |  |  |  |  |  |
| I need additional support to complete the job I am expected to do as a treatment partner for HIV |  |  |  |  |  |  |
| I am expected to do too many things as a treatment partner for HIV |  |  |  |  |  |  |

**SECTION 3: High Blood Pressure (HTN) Care**

16. For the following section, think about your role as a treatment partner. As a treatment partner for high blood pressure, read the statement and inform us if you: Strongly agree, Agree, Neither Agree or Disagree, Disagree, Strongly Disagree

|  | Strongly Disagree | Disagree | Neither Agree nor Disagree | Agree | Strongly Agree | No Answer |
| --- | --- | --- | --- | --- | --- | --- |
| Combining high blood pressure and HIV care in the same clinic visit will improve the blood pressure of my peer |  |  |  |  |  |  |
| Including treatment partners to help patients manage both HIV and high blood pressure would be too complicated |  |  |  |  |  |  |
| A treatment partner for high blood pressure will help patients remember to go to appointments. |  |  |  |  |  |  |
| A treatment partner for high blood pressure will help patients remember to take their medications. |  |  |  |  |  |  |
| A treatment partner will be able to teach patients about high blood pressure and how to manage their condition |  |  |  |  |  |  |
| Treatment partners will be successful in helping patients make changes to their diet to reduce blood pressure |  |  |  |  |  |  |
| Treatment partners will be successful in helping patients increase their physical activity to reduce blood pressure |  |  |  |  |  |  |

Score *Note. 1 = Strongly Disagree, 2 = Disagree, 3 = Neither Agree nor Disagree, 4 = Agree, 5 = Strongly Agree*.

*Items were reverse scored prior to conducting t-tests and creation of average score

**Health Care Provider Survey**

**Broader clinic view**

1. Gender: a) Female b) Male c) Other d) Refuse to answer

2. Age……………………………

3. Role at Health Facility

- - - - 1. Medical Officer
        2. FNP
        3. Nurse
        4. Dietician
        5. Pharmacist
        6. Other (please specify) …………….

4. Highest Academic Qualification: ……………………………………………

5. Years working at the Clinic………………………………………….

6. List on-job Hypertension training you received in past 2 years.

7. On a scale of 1-5 (1 not confident, 5 being very confident), how confident do you feel:

|  | **1** | **2** | **3** | **4** | **5** |
| --- | --- | --- | --- | --- | --- |
| Diagnosing hypertension (HTN) |  |  |  |  |  |
| Prescribing hypertension medications |  |  |  |  |  |
| Counseling patients on diet for hypertension |  |  |  |  |  |
| Identifying when hypertension is not well controlled |  |  |  |  |  |
| Adjusting medications when hypertension is not controlled |  |  |  |  |  |

8. How many HIV Positive patients do you care for that also have hypertension?

1. None
2. 1-10 Patients a week
3. 11-30 Patients a week
4. 31-50 Patients a week
5. Over 50 Patients a week

9. Given how you understand InterCARE, how important do you think each component is (from not all important to very important)

| **InterCARE Component** | Not at all Important | Slightly  Important | Important | Fairly  Important | Very Important |
| --- | --- | --- | --- | --- | --- |
| Training |  |  |  |  |  |
| Electronic Health Records (EHR) |  |  |  |  |  |
| Treatment partner |  |  |  |  |  |

Score *Note. 1 = Not at all Important, 2 =Slightly Important, 3 = Important, 4 = Fairly Important, 5=Very Important*

Attitudes for Adoption

| 10. | Strongly Disagree | Disagree | Neither Agree nor Disagree | Agree | Strongly Agree |
| --- | --- | --- | --- | --- | --- |
| 10. InterCARE would be *more* effective than interventions we are currently using to manage HTN in PLWH in my clinic |  |  |  |  |  |
| *11. InterCARE is* too complex to put into place in my clinic.* |  |  |  |  |  |
| *12. InterCARE* would be successful in improving treatment of HIV-positive individuals with HTN in my clinic |  |  |  |  |  |
| *13. InterCARE* is compatible and consistent with the needs of HIV-positive individuals with HTN in my clinic |  |  |  |  |  |
| *14. InterCARE* requires too many staff or other resources. * |  |  |  |  |  |
| *15. InterCARE* would be easy to understand and use after receiving training. |  |  |  |  |  |
| *16. InterCARE* would have a visible and substantial impact on the health status of HIV-positive individuals with HTN in my clinic |  |  |  |  |  |
| 17. HIV-positive individuals with HTN in my clinic would really benefit from *InterCARE* |  |  |  |  |  |
| 18. It would be difficult to adapt *InterCARE* to meet the needs of different populations/groups pf HIV-positive individuals with HTN in my clinic |  |  |  |  |  |
| *19. InterCARE* would be problematic because we do not have enough HIV medical and supportive care resources to care for any additional HIV-positive patients with HTN |  |  |  |  |  |

Score *Note. 1 = Strongly Disagree, 2 = Disagree, 3 = Neither Agree nor Disagree, 4 = Agree, 5 = Strongly Agree*.

*Items were reverse scored prior to conducting t-tests and creation of average score.

**End of Pre-Implementation Survey.**

**Community Member Survey**

Goal of the survey:

- Understand the experience of community related to HIV and HTN
- Understand what they view as areas for improvement in HTN management (may or may not be related to InterCARE)
- Explore their attitudes towards integrating Mopati for HIV and HTN

SECTION 1: Baseline Information

1. Age……………….

Gender: a. Female b) Male c) Other d) Refuse to answer

What is your role in the community?

a. Community leader

b. Consumer Services sector

c. Local Counsel

4. Highest level of school completed

- 1. Less than Primary (Including Non formal)
  2. Primary School (Standard 1-7)
  3. Junior Secondary (Form 1-3)
  4. Senior Secondary (Form 4-5)
  5. Higher than senior secondary (university, diploma, etc.)

5. What is the main source of energy used for cooking?

a. Charcoal/wood

b. Paraffin

c. Gas

d. Electricity (mains)

e. Electricity (solar)

f. No cooking done

g. Other

6. What is the main toilet facility used in this household?

a. Pit latrine within plot

b. Flush toilet within plot

c. Neighbor’s flush toilet

d. Neighbor’s pit latrine

e. Communal flush toilet

f. Communal pit latrine

g. Pail bucket latrine

h. Bush

i. River or other body of water

j. Other

7. Does any member of this household have any of the following that are currently working? (check all that apply).

a. Radio

b. TV

c. Landline telephone

d. Cell phone

e. Computer

f. Access to internet

g. Refrigerator

h. Don't want to answer

8. Does any member of this household (excluding visitors) own any of the following forms of transport in working condition? (check all that apply).

a. Motor vehicle (car, truck, taxi, etc)

b. Tractor

c. Bicycle

d. Motorcycle/scooter

e. Donkey or cow cart

f. Donkey/horses

9. Are you currently working?

1. Yes – Question 4
2. No – Question 5

10. In your main job what type of work do you do?

1. Occasional or Casual employment (piece job)
2. Seasonal employment
3. Formal wage employment (full-time)
4. Formal wage employment (part-time)
5. Self-employed in agriculture
6. Self-employed making money, full time
7. Self-employed making money, part time
8. Other

11. What is the reason why you are not working?

1. Waiting to continue agricultural work
2. Unemployed (looking for work)
3. Unemployed (waiting to start new work)
4. Unable to work (permanently sick or injured)
5. Student/ Apprentice/ Volunteer
6. Housewife/ Homemaker (not looking for work)
7. Retired
8. Other
9. Not looking for work

12. Monthly Household Income

a) < 1000

b) 1001 – 5000

c) 5001 - 10,000

d) 10,001 & above

e) Prefer not to answer

f) I don’t know

13. Number of members (including participant) living in the household? __________

14. When was the last time you visited this clinic?

1. Never
2. Less than 30 days ago
3. 1-6 Months back
4. 7-12 Months back
5. More than 12 months back
6. I don’t know

| 15 | Strongly Disagree | Disagree | Neither Agree nor Disagree | Agree | Strongly Agree |
| --- | --- | --- | --- | --- | --- |
| 1. Combining high blood pressure and HIV care in the same clinic visit will improve a patient’s blood pressure |  |  |  |  |  |
| 1. Having a peer in the community to help patients manage both my HIV and high blood pressure would be too complicated |  |  |  |  |  |
| 1. Combining high blood pressure and HIV care into the same clinic visit would be too complicated |  |  |  |  |  |
| 1. Having a treatment partner will help patients remember to go to their appointments |  |  |  |  |  |
| 1. Having a treatment partner will help patients remember to take their medications for high blood pressure |  |  |  |  |  |
| 1. Having a treatment partner will help patients make changes to their diet to reduce blood pressure |  |  |  |  |  |
| 1. Having a treatment partner will help patients increase their physical activity to reduce blood pressure |  |  |  |  |  |
| 1. This program will be successful in improving patient knowledge about managing their only high blood pressure |  |  |  |  |  |

Score *Note. 1 = Strongly Disagree, 2 = Disagree, 3 = Neither Agree nor Disagree, 4 = Agree, 5 = Strongly Agree*.

*Items were reverse scored prior to conducting t-tests and creation of average score.

**END OF SURVEY**
