# Supplementary material for: A mixed methods approach identifying facilitators and barriers to guide adaptations to InterCARE strategies: an integrated HIV and hypertension care model in Botswana
Source: Implement Sci Commun. 2024 Jun 20;5:67. doi: 10.1186/s43058-024-00603-x (PMC11188218; doi:10.1186/s43058-024-00603-x)
Supplement: Supplementary file 2 — Supplementary Material 2. [file 43058_2024_603_MOESM2_ESM.docx]

COREQ Checklist for Qualitative Data Reporting

| DOMAIN 1: Research Team and Reflexivity |  | | Page  Number |  |
| --- | --- | --- | --- | --- |
| 1. Interviewer/facilitator | Which author/s conducted the interview or focus groups? | | 10 | Research assistants |
| 1. Credentials | What were the researcher’s credentials | | 10 | “University-educated research assistants fluent in both the local language, Setswana, and in English” |
| 1. Occupation | What was their occupation at the time of the study | | 10 | Research assistants |
| 1. Gender | Was the researcher male or female? | | 9 | Three female, one male |
| 1. Experience and training | What experience or training did the researcher have? | | 10 | “…underwent survey administration and qualitative research training.” |
| Relationship with participants |  | |  |  |
| 1. Relationship | Was a relationship established prior to study commencement. | | 10 | “KIIs were conducted anonymously by the same research assistants who had no prior relationships with the participants.” |
| 1. Participant knowledge of the interviewer | What did the participants know about the researcher? E.g., personal goals, reasons for doing the research | | 10 | “KIIs were conducted anonymously by the same research assistants who had no prior relationships with the participants.” |
| 1. Interviewer characteristics | What characteristics were reported about the interviewer/facilitator | | 9 | Reported gender of trained research assistants |
| Domain 2: Study Design |  | |  |  |
| 1. Methodological orientation and theory | What methodological orientation was stated to underpin the study | | 11-12 | Direct content analysis |
| Participant selection |  | |  |  |
| 1. Sampling | How were participants selected? E.g., purposive, convenience | | 8 | “For key informant interviews (KIIs), a diverse sample of stakeholders with a wide range of perspectives were purposively selected based on their clinic location, and availability to be interviewed (n=2 HCWs, n=3 community members, n=2 treatment partners, n=3 participants) (Table 2, Supplemental Materials).” |
| 1. Method of approach | How were participants approached? E.g., face-to-face, telephone, mail, email | | 8 | Participants were approached at HIV clinic.  “PLWH and HTN (n=20, 50% female), HIV treatment partners (n=20, 95% female) and HCWs (n=20, 55% female) were consecutively recruited in person at each clinic site for the pre-implementation surveys and community members (n=40, 57.5% female) were purposively selected based on role in the community until the targeted number was reached (Table 1, Supplemental Materials). For key informant interviews (KIIs), from the stakeholders surveyed, a diverse sample of stakeholders with a wide range of perspectives were purposively selected based on their clinic location, and availability to be interviewed (n=2 HCWs, n=3 community members, n=2 treatment partners, n=3 participants) (Table 2, Supplemental Materials).” |
| 1. Sample size | How many participants were in the study? | | 8 | 10 participants (n=2 healthcare providers, n=3 community members, n=2 treatment partners, n=3 patients) |
| 1. Non-participation | How many people refused to participate or dropped out? Reasons? | | 8 | There were no refusals or drop outs for qualitative interviews. |
| Setting |  | |  |  |
| 1. Setting of data collection | Where was the data collection? E.g., home, clinic, workplace | | 10 | “KIIs were conducted…in a private room in the clinic” |
| 1. Prescence of non-participants | Was anyone else present besides the participants and researchers? | | 10 | No other individuals were present |
| 1. Description of sample | What are the important characteristics of the sample? E.g., demographic data, date | | Supplemental Materials, Table 1 | See Supplemental Materials, Table 1 |
| Data collection |  | |  |  |
| 1. Interview guide | Were questions, prompts, guides provided by the authors? Was it pilot tested? | | 9, 10 | “Semi-structured KIIs were developed guided by CFIR and other factors (e.g., HIV stigma) known to affect HIV care from prior research. KIIs were intended to explore stakeholders’ understanding of and experiences with HTN, challenges managing HTN in the current system, and perceptions of the InterCARE intervention (acceptability, feasibility, and relative advantage) (Table 4, Supplemental Materials).”  “These trained research assistants pilot tested surveys and KIIs on all key stakeholder groups for feasibility and readability.” |
| 1. Repeat interviews | Were repeat interviews carried out? If yes, how many? | | 10 | No repeat interviews were carried out |
| 1. Audio/visual recording | Did the research use audio or visual recording to collect the data? | | 10f | Interviews were audio-recorded, transcribed, and translated to English where necessary. |
| 1. Field notes | Were field notes made during and/or after the interview of focus group? | | 10 | No field notes were made during the interviews |
| 1. Duration | What was the duration of the interviews or focus group? | | 10 | “…approximately 30 minutes” |
| 1. Data saturation | Was data saturation discussed? | | 8, 10 | A set number of interviews was planned, no protocol to collect data until data saturation |
| 1. Transcripts returned | Were transcripts returned to participants of comment/or correction? | | 10 | Transcripts were not returned to participants for comment or correction |
|  | | Domain 3: analysis and findings | | |
|  | | Data analysis | | |
| 1. Number of data coders | How many data coders coded the data? | | 11, 12 | “Two investigators (PG, NY) read all of the transcripts, and independently manually coded the same two full transcripts in Microsoft Word to identify preliminary codes” |
| 1. Description of the coding tree | Did authors provide a description of the coding tree? | | Supplemental Materials, Table 4 | Supplemental Materials, Table 4 |
| 1. Derivation of themes | Were themes identified in advance or derived from the data? | | 12 | “After discussion of these codes and use of consensus strategies to resolve disagreements, an initial codebook was created and applied to transcripts. In subsequent meetings, a final codebook was agreed upon, and subthemes were mapped onto the updated CFIR domains and constructs.^28^ Coding and subthemes were reviewed by an additional investigator, and both investigators met with senior investigators to reach consensus.” |
| 1. Software | What software, if applicable, was used to manage the data? | | 12 | Microsoft Word |
| 1. Participant checking | Did participants provide feedback on the findings? | | 12 | No, participants did not provide feedback on the findings |
| Reporting |  | |  |  |
| 1. Quotations presented | Were participant quotations presented to illustrate the themes/findings? Was each quotation identified? E.g., participant number | | 13-19 | Quotations to illustrate themes and findings were embedded in the document |
| 1. Data and findings consistent | Was there consistence between the data presented and the findings? | | 12 | There was consistency between the data presented and the findings. Coding and subthemes were reviewed by an additional investigator and both investigators met with senior investigators to reach consensus and resolve disagreements. |
| 1. Clarity of major themes | Were major themes clearly presented in the findings? | | 13-10 | Major themes under the codes of CFIR constructs were clearly presented |
| 1. Clarity of minor theme | Is there a description of diverse cases of discussion of minor themes? | | 13-19, Supplemental Materials Table 7 | Multiple minor themes were included in the documentation |
